# Insights into the correlation between Physiological changes in and seed development of tartary buckwheat (*Fagopyrum tataricum* Gaertn*.*)

**DOI:** 10.1186/s12864-018-5036-8

**Published:** 2018-08-31

**Authors:** Moyang Liu, Zhaotang Ma, Tianrun Zheng, Wenjun Sun, Yanjun Zhang, Weiqiong Jin, Junyi Zhan, Yuntao Cai, Yujia Tang, Qi Wu, Zizhong Tang, Tongliang Bu, Chenglei Li, Hui Chen

**Affiliations:** College of Life Science, Sichuan Agricultural University, Ya’an, China

**Keywords:** nutrition, phytohormones, RNA sequencing (RNA-seq), seed development, tartary buckwheat, transcriptome

## Abstract

**Background:**

Tartary buckwheat *(Fagopyrum tataricum* Gaertn.) is a widely cultivated medicinal and edible crop with excellent economic and nutritional value. The development of tartary buckwheat seeds is a very complex process involving many expression-dependent physiological changes and regulation of a large number of genes and phytohormones. In recent years, the gene regulatory network governing the physiological changes occurring during seed development have received little attention.

**Results:**

Here, we characterized the seed development of tartary buckwheat using light and electron microscopy and measured phytohormone and nutrient accumulation by using high performance liquid chromatography (HPLC) and by profiling the expression of key genes using RNA sequencing with the support of the tartary buckwheat genome. We first divided the development of tartary buckwheat seed into five stages that include complex changes in development, morphology, physiology and phytohormone levels. At the same time, the contents of phytohormones (gibberellin, indole-3-acetic acid, abscisic acid, and zeatin) and nutrients (rutin, starch, total proteins and soluble sugars) at five stages were determined, and their accumulation patterns in the development of tartary buckwheat seeds were analyzed. Second, gene expression patterns of tartary buckwheat samples were compared during three seed developmental stages (13, 19, and 25 days postanthesis, DPA), and 9 765 differentially expressed genes (DEGs) were identified. We analyzed the overlapping DEGs in different sample combinations and measured 665 DEGs in the three samples. Furthermore, expression patterns of DEGs related to phytohormones, flavonoids, starch, and storage proteins were analyzed. Third, we noted the correlation between the trait (physiological changes, nutrient changes) and metabolites during seed development, and discussed the key genes that might be involved in the synthesis and degradation of each of them.

**Conclusion:**

We provided abundant genomic resources for tartary buckwheat and Polygonaceae communities and revealed novel molecular insights into the correlations between the physiological changes and seed development of tartary buckwheat.

**Electronic supplementary material:**

The online version of this article (10.1186/s12864-018-5036-8) contains supplementary material, which is available to authorized users.

## Background

Tartary buckwheat (*Fagopyrum tataricum* Gaertn.) comprises twenty different species of extensively cultivated medicinal and edible crop species with excellent economic and nutritional values [1, 2]. Tartary buckwheat was domesticated in East Asia and is cultivated in Europe and North America [3]. Today, it is common in the Himalayan region and Southwest China, such as Sichuan Province. In 2016, global production was 2.3 million tons, with Russia and China accounting for 50% and 17% of the world’s total output, respectively (adopted from UN Food and Agriculture Organization, Corporate Statistical Database: http://www.fao.org/). Tartary buckwheat seeds are a rich source of four B vitamins, dietary fiber, protein and a variety of minerals, in which niacin, magnesium, manganese, phosphorus content is particularly high (adopted from USDA Food Composition Databases: https://ndb.nal.usda.gov/). Importantly, tartary buckwheat seeds are known for their high rutin content, which has been proven to be effective in preventing liver injury and especially inflammatory liver injury [4].

The development of most seeds can be divided into three stages: tissue differentiation, cell enlargement and mature dehydration. At the stage of tissue differentiation, a single-cell zygote forms a young embryo composed of cotyledons by cell division and differentiation [5, 6]. Seed development in higher plants is a very complex process involving many expression-dependent physiological changes and regulation of a large number of genes and phytohormones, and the development of tartary buckwheat seeds is no exception.

Although the regulatory networks of monocotyledonous plants (i.e. maize) seed development [7] and various nutritional and pharmacological effects of tartary buckwheat have been well studied [8–11], there are few reports on the gene regulatory network governing the physiological changes occurring during seed development of tartary buckwheat of dicotyledonous plants supported by the tartary buckwheat genome. Here, we characterized the seed development of tartary buckwheat using light and electron microscopy, quantification of phytohormone and nutrient accumulations using HPLC, and by profiling the expression of key genes through transcriptome profiling. By analyzing the correlation between phytohormone changes and seed development, we investigated embryo cell enlargement and primary dormancy of tartary buckwheat seeds. By analyzing the correlation between nutrient changes and seed development, we determined the gene regulatory network controlling the accumulation of rutin, starch, storage protein and soluble sugars during the development of tartary buckwheat seeds and analyzed the key genes. We provided abundant genomic resources for tartary buckwheat and Polygonaceae communities and revealed novel molecular insights into the correlations between the physiological changes and seed development of tartary buckwheat.

## Results

### In-depth description of the development of tartary buckwheat seed

**Fig. 1:**
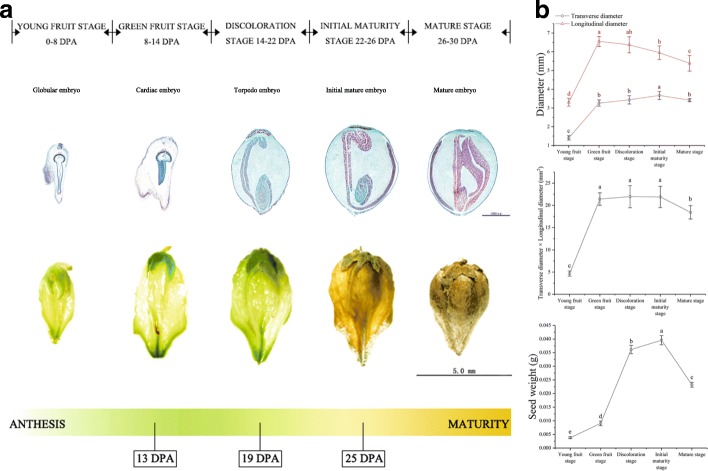
Observation and measurement of the development of tartary buckwheat seeds. **a** Five stages of tartary buckwheat seeds development. Top row shows each longitudinal sections of tartary buckwheat seeds at different developmental stages. Second row shows individual seeds at different developmental stages. Third row shows schematic representation of developmental stages studied during tartary buckwheat seed ontogeny. 13 DPA: Tartary buckwheat seed at green fruit stage for transcriptome experiment; 19 DPA: Tartary buckwheat seed at discoloration stage for transcriptome experiment; 25 DPA: Tartary buckwheat seed at initial maturity stage for transcriptome experiment. DPA, days-post-anthesis. **b** The seed size and weight at different developmental stages. Error bars were obtained from five measurements. Small letter(s) above the bars indicate significant differences (α = 0.05, LSD) among treatments

The development of tartary buckwheat seed from anthesis to maturation was divided into five stages that include complex changes in development, morphology, physiology and phytohormone levels (Fig. 1a, b). The young fruit stage was 0-8 DPA, and the globular embryo gradually formed. At this time, the ovary cavity of the fruit was larger, and the seed was smaller, presenting light green coloration with deep grooves. During 8-14 DPA in the green fruit stage, the heart-shaped embryo formed in steps, the proportion of seeds in the fruit increased, the color of the fruit deepened, and the grooves became shallower. It is worth mentioning that the longitudinal diameter of the fruit reached the maximum at this time. At the discoloration stage (14-22 DPA), the fruit size reached the largest, the green color was the deepest, and the grooves were not obvious; the torpedo-shaped embryo also formed at this time. At the initial maturity stage (22-26 DPA), fruit weight reached the maximum, and the fruit began to show signs of water loss, turn yellow in color, became smaller in size, and developed deeper grooves. The mature stage was 26-30 DPA, during which time the color of the seed turned dark yellow, the seed filled up the entire fruit, and the cotyledon changed from transparent to white, showing the maturity of the embryo. The shape of the developing embryo in each developing seed is a more reliable time marker [12]. Based on the number of days postanthesis, the young fruit stage to maturity stage described here correspond to the globular embryo to the mature embryo in tartary buckwheat seeds.

### Phytohormone accumulation at different stages of seed development

**Fig. 2:**
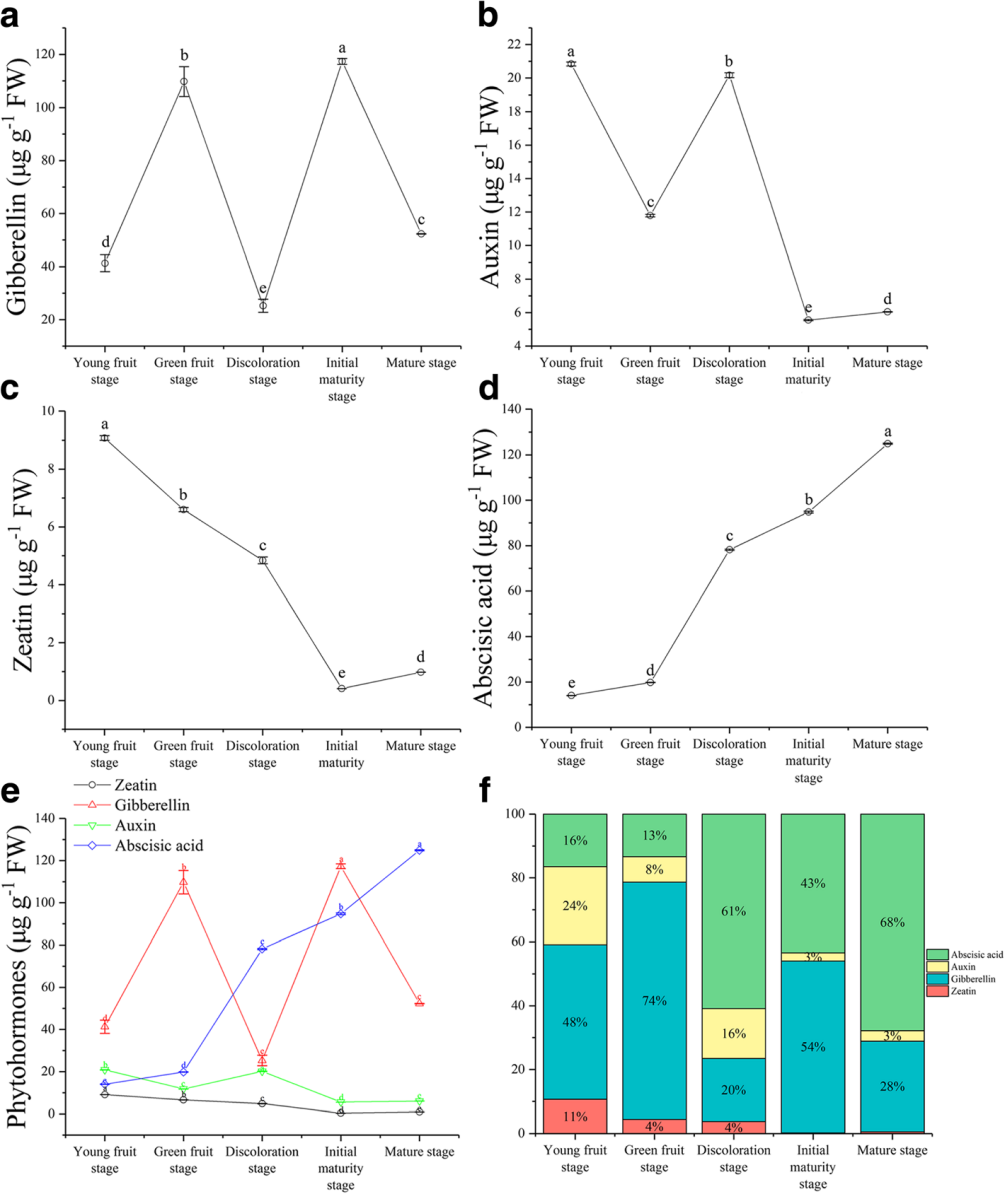
Changes in the levels of GA_3,_ IAA, ZT, and ABA during seed development in tartary buckwheat. **a-d** Changes of GA_3,_ IAA, ZT, and ABA content during seed development in tartary buckwheat, respectively. **e** GA_3_ (red line), IAA (green line), ZT (black line), and ABA (blue line) content at different developmental stages. **f** Phytohormone ratio during seed development in tartary buckwheat. Error bars were obtained from three measurements for **(a)**, **(b)**, **(c)**, **(d)** and **(e)**. Small letter(s) above the bars indicate significant differences (α = 0.05, LSD) among treatments

In buckwheat seeds, the content of gibberellin (GA_3_) was at a high level during the young fruit stage and peaked at 13 DPA with a value of 109.76 μg g^−1^ fresh weight (FW). After a sharp reduction until the discoloration stage, the content of GA_3_ decreased to the lowest value of 25.28 μg g^−1^ FW. The second increase occurred before the initial maturity stage and reached a maximum of 117.34 μg g^−1^ FW at 19 DPA. Throughout the development process, the GA_3_ content appeared as a fluctuation curve (Fig. 2a). The level of indole-3-acetic acid (IAA) also appeared as a fluctuation curve. The IAA content was high at young fruit stage and decreased sharply at green fruit stage. The second increase occurred before the discoloration stage and reached a value of 20.17 μg g^−1^ FW at 19 DPA, after which it decreased again from this time to the initial maturity stage, with the lowest value of 5.56 μg g^−1^ FW. After a sharp reduction, it started to increase again to a content of 6.05 μg g^−1^ FW (Fig. 2b). The content of zeatin (ZT) was at a high level during the young fruit stage and reached a peak value of 9.08 μg g^−1^ FW, after which it decreased sharply before the initial maturity stage. The content of ZT decreased to the lowest value of 0.41 μg g^−1^ FW, but after a sharp reduction before the maturity stage, it started to rise again to a content of 0.98 μg g^−1^ FW. Throughout the development process, the ZT content remained at a low level (Fig. 2c). The abscisic acid (ABA) content increased sharply and reached a peak value of 124.93 μg g^−1^ FW at the maturity stage (Fig. 2d). The peak of these three hormones in the process of seed development appeared in the order of ZT, IAA, GA_3_, and ABA (Fig. 2e). Among the four phytohormones, their proportions were different at different stages of seed development (Fig. 2f).

### Nutrient accumulation at different stages of seed development

**Fig. 3:**
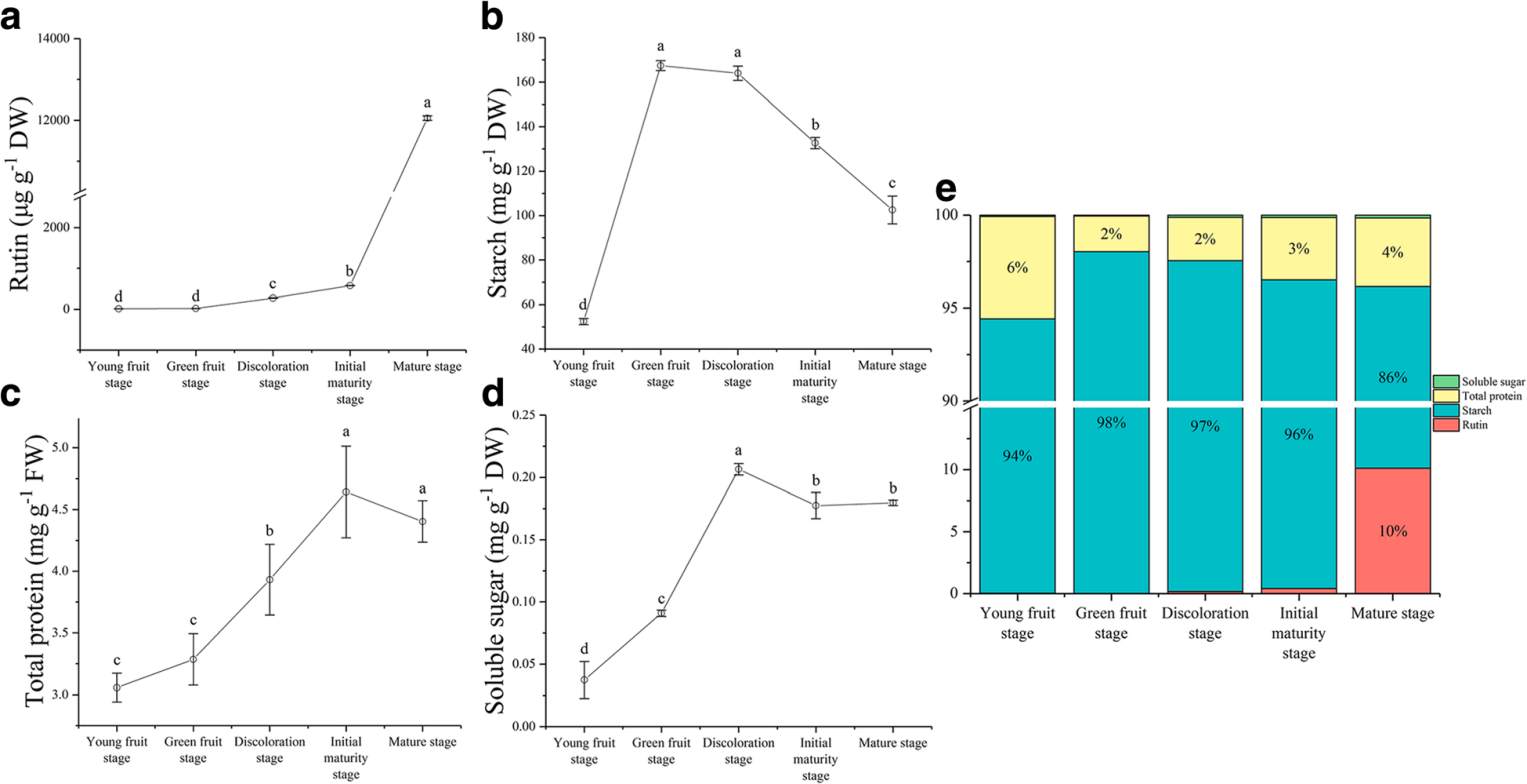
Changes in the levels of nutrients at different development stages of tartary buckwheat seed. **a-d** Changes of rutin, starch, total protein, and soluble sugar content during seed development in tartary buckwheat, respectively. **e** Nutrients ratio during seed development in Tartary buckwheat. The error bars were obtained from five measurements. Small letter(s) above the bars indicate significant differences (α = 0.05, LSD) among treatments

As shown in Fig. 3, the four following components were quantified, namely, rutin, starch, total proteins and soluble sugars. The content of rutin increased linearly after anthesis and notably reached a peak value of 12 055.78 μg g^−1^ dry weight (DW) at the mature stage (Fig. 3a). The content of starch rapidly increased during the transition to green fruit stage and reached a maximum of 167.40 mg g^−1^ DW, after which it decreased linearly to a content of 102.53 mg g^−1^ DW (Fig. 3b). The content of total protein gradually increased after anthesis, notably from the discoloration stage to the initial maturity stage, and reached a maximum of 4.64 mg g^−1^ FW at the initial maturity stage, after which it started to drop rapidly to a content of 4.40 mg g^−1^ FW (Fig. 3c). The soluble sugar linearly increased from the young fruit stage to the discoloration stage and reached a peak value of 0.21 mg g^−1^ DW at the discoloration stage. After a slight drop, it started to rise again to a content of 0.18 mg g^−1^ DW (Fig. 3d). Among the four nutrients, starch dominated with relatively high proportions at the different stages of seed development (Fig. 3e).

### Correlation between physiological and developmental changes in tartary buckwheat seeds

**Fig. 4:**
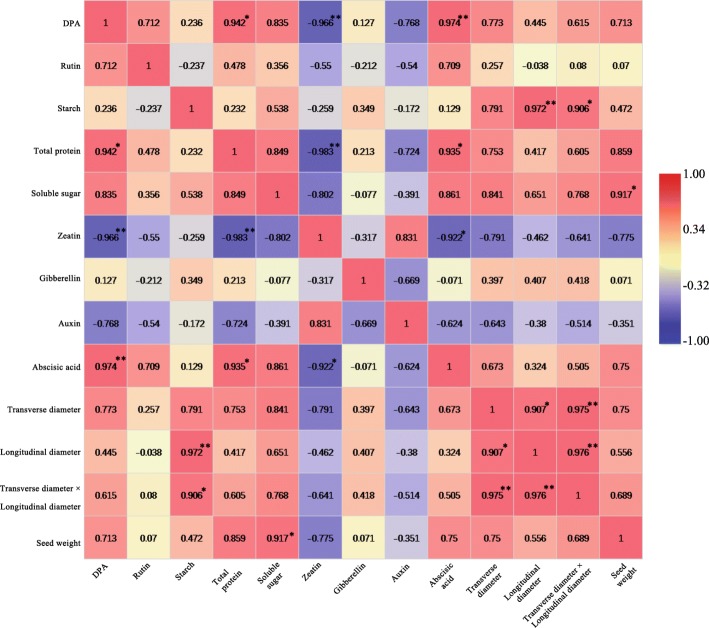
The correlation between physiological changes and seed maturity during seed development (7 DPA to 30 DPA). Digit: Pearson’s correlation coefficient; Red: positively correlated; Blue: negatively correlated. * and ** indicate significant correlation at 0.05 and 0.01 levels, respectively

The linear relationship between seed maturity and physiological changes were analyzed. The results showed that total protein, ZT and ABA were significantly correlated with seed maturity, and the absolute values of the correlation coefficients were all greater than 0.94 (Fig. 4). Seed size (transverse diameter × longitudinal diameter) was positively related to starch, with a correlation coefficient value of 0.906. Longitudinal diameter was significantly related to starch, and the correlation coefficient was 0.972. Seed weight was positively related to soluble sugar, with a correlation coefficient value of 0.917 (Fig. 4). Total protein was significantly related to ABA and ZT, and the absolute values of the correlation coefficients were greater than 0.93. The total protein was positively related to ABA and was negatively related to ZT. ZT was negatively related to ABA, and the absolute value of the correlation coefficient was 0.922. However, rutin, GA_3_, IAA, seed size, seed weight and DPA were not significantly correlated (Fig. 4).

### Transcriptomic profiles during seed development

**Fig. 5:**
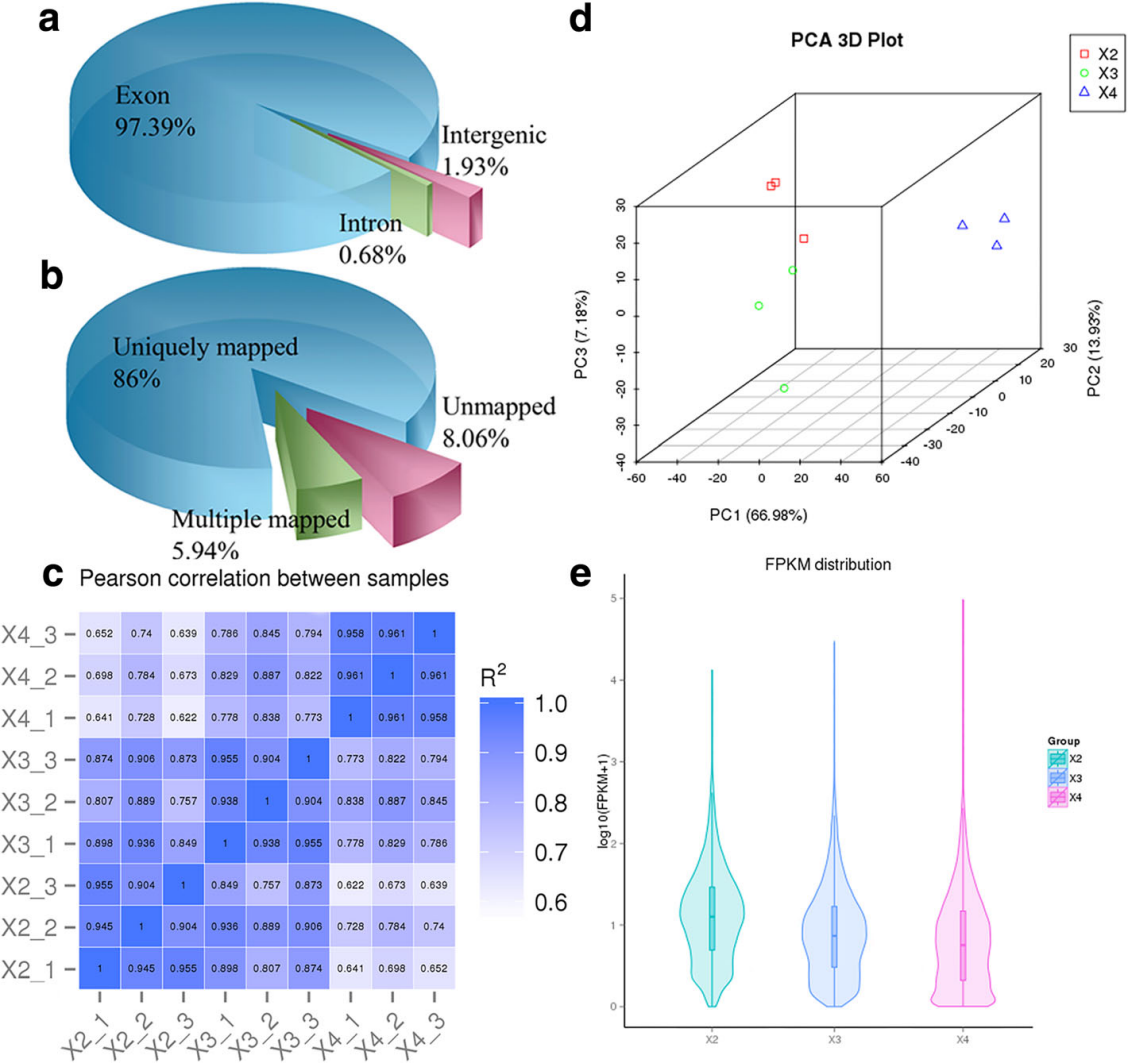
Distribution and quality of reads in different samples. **a** Distribution of overall mapped reads for all samples in the different regions. **b** Overall results of paired-end reads (PEs) for all samples mapped to the reference genome. **c** Correlation between samples. **d** Principal component analysis (PCA) among biological replicates. **e** Gene expression distribution (FPKM) in three samples

To comprehensively assess the change in the transcript profile, we analyzed RNAs from three seed developmental stages (13, 19, and 25 DPA). The raw reads of 9 samples ranged from 51 to 63 million, with an error rate of approximately 0.02, resulting in 7.61–8.84 G of clean bases (Additional file 1: Table S1). In exon region, intron region and intergenic region, the reading rates of mapping were 97.4, 0.68, and 1.93%, respectively (Fig. 5a). Approximately 91.94% of total reads were mapped to the reference genome. Among them, the unique map reads accounted for 86.0% of the total reads, and multiple map reads accounted for 5.94% (Fig. 5b, Additional file 1: Table S2). The overall quality of the RNA-seq data was evaluated by correlation analysis between samples and principal component analysis (PCA) of biological replicates (Fig. 5d). The correlation coefficients between all biological replicate samples were greater than 0.90 (Fig. 5c, Additional file 1: Table S3). Three biological replicates, especially at 19 DPA, were significantly clustered, indicating that the expression patterns in the sample had high similarity and that the sequencing data could be used to analyze the differential expression of the genes. The expression levels of the genes were estimated by the fragments per kilobase of transcripts per million mapped fragments (FPKM) [13]. The level of gene expression showed a similar trend among the three samples, and the expression level of most genes was relatively low (Fig. 5e).

### Analysis of differentially expressed genes during seed development

**Fig. 6:**
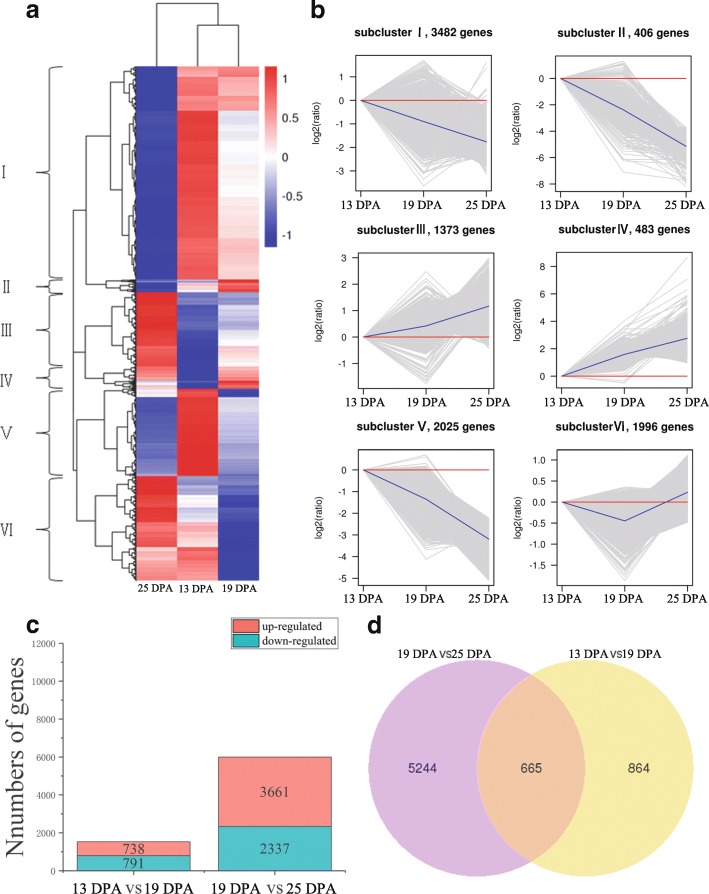
DEGs in three samples. **a** Heat map of scaled FPKM values in three samples 13 DPA, 19 DPA, and 25 DPA. Red: high expression; Blue: low expression. **b** Expression profile of six clusters correspondance to the Hierarchical cluster result. **c** Number of up- and down-regulated DEGs in two sample pairs 13 DPA vs 19 DPA, and 19 DPA vs 25 DPA. **d** Venn diagram of the numbers of expressed genes in sample pairs 13 DPA vs 19 DPA, and 19 DPA) vs 25 DPA

To understand gene expression during tartary buckwheat seed development (13, 19, and 25 DPA), we compared and analyzed the DEGs in two pairs of samples (13 DPA vs 19 DPA, 19 DPA vs 25 DPA). A total of 9 765 DEGs were divided into 6 groups (Fig. 6a). Similarly, they could be divided into two broad categories according to their expression patterns. The first category contained groups III, IV and VI, with upregulated genes. The second category contained groups I, II, and V, in which the genes showed downregulation patterns. In detail, groups III and IV showed a gradual upward trend during the seed development stage. The genes in group VI were upregulated at 25 DPA compared with those at 13 DPA and 19 DPA. Groups I, II and V showed a decreasing trend at the stage of seed development (Fig. 6b). We found significant differences in gene expression during seed development and detected 1 529 DEGs (791 downregulated and 738 upregulated) at 13 DPA vs 19 DPA and 5 998 DEGs (2 337 downregulated and 3 661 upregulated) at 19 DPA vs 25 DPA (Fig. 6c). Besides, the expression profiles of 20 genes in the 13DPA, 19DPA, and 25 DPA, based on RT-qPCR, were consistent with the RNA-seq results with a Pearson correlation coefficient of 0.31 (P < 0.05) (Additional file 1: Table S6). To characterize the expression pattern of genes during tartary buckwheat seed development, we analyzed the overlapping DEGs in different sample combinations and measured 665 DEGs in the three samples (Fig. 6d); in the comparisons of 13 DPA vs 19 DPA and 19 DPA vs 25 DPA, there were 864 and 5 244 DEGs, respectively (Fig. 6d).

### Expression patterns of DEGs related to phytohormones during seed development

**Fig. 7:**
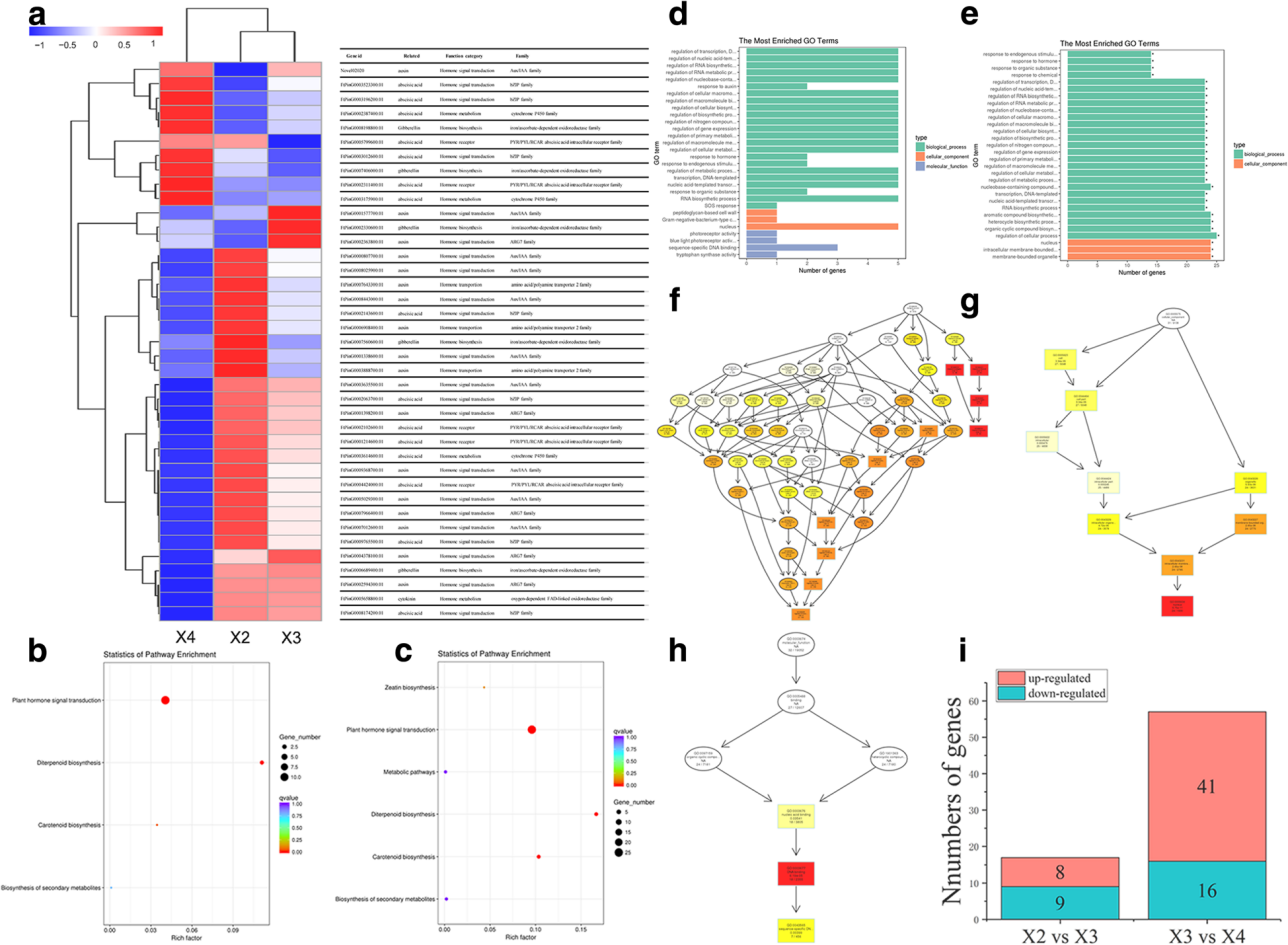
DEGs related to phytohormones in three samples. **a** Hierarchical cluster of the DEGs related to phytohormones in X2 (13 DPA), X3 (19 DPA), and X4 (25 DPA). Red: high expression; Blue: low expression. **b** Scatterplot of KEGG pathway enrichment in X2 (13 DPA) vs X3 (19 DPA) (FDR< 0.05). **c** Scatterplot of KEGG pathway enrichment in X3 (19 DPA) vs X4 (25 DPA) (FDR< 0.05). Rich factor is the ratio of the number of DEGs to the number of background genes in a KEGG pathway. **d** GO classification of DEGs in X2 (13 DPA) vs X3 (19 DPA) (FDR< 0.05). **e** GO classification of DEGs in X3 (19 DPA) vs X4 (25 DPA) (FDR< 0.05). The top 30 enriched GO classifications are listed. Stars above bars indicate the amounts of differentially expressed genes are significantly higher or lower than the amounts of genes in random samples from the GO classification of all genes. **f** DEGs related to phytohormones enriched biological processes in X3 (19 DPA) vs X4 (25 DPA). **g** DEGs related to phytohormones enriched cellular component in X3 (19 DPA) vs X4 (25 DPA). **h** DEGs related to phytohormones enriched molecular function in X3 (19 DPA) vs X4 (25 DPA). The different color frames indicate the extent of significance. Yellow: significant; Red: extremely significant. **i** Number of up- and down-regulated DEGs in two sample pairs X2 (13 DPA) vs X3 (19 DPA), and X3 (19 DPA) vs X4 (25 DPA)

Phytohormones affect gene expression and transcription levels, cellular division, and plant growth [14]. Therefore, we identified the DEGs related to phytohormones. The results showed that 277 genes were associated with four major phytohormones, including ABA (35 genes), IAA (185 genes), ZT (20 genes) and GA_3_ (37 genes) (Additional file 1: Table S4). These genes are related to all aspects of phytohormone homeostasis, including biosynthesis (33 genes), metabolism (15 genes), receptors (26 genes), responses (3 genes), signal transduction (178 genes) and transportation (22 genes) (Table 1). The number of them found in a complex regulatory network of seed development are presented on Fig. 7a. We detected 17 DEGs (9 downregulated DEGs and 8 upregulated DEGs) in 13 DPA vs 19 DPA and 57 DEGs (16 downregulated DEGs and 41 upregulated DEGs) in 19 DPA vs 25 DPA (Fig. 7i). In 13 DPA vs 19 DPA, the DEGs were mostly enriched in 4 KEGG pathways in the KEGG database (False discovery rate (FDR) < 0.05) (Fig. 7b). However, a Gene Ontology (GO) analysis indicated that DEGs were not significantly enriched (FDR < 0.05) (Fig. 7d). In 19 DPA vs 25 DPA, the DEGs were mostly enriched in 6 KEGG pathways in the KEGG database (FDR < 0.05) (Fig. 7c). A GO analysis indicated that 27 terms involving biological process (Fig. 7e), 9 terms involving cellular component (Fig. 7f), and 3 terms involving molecular function (Fig. 7h) were significantly enriched (FDR < 0.05) (Fig. 7e).

**Table 1:** Genes related to phytohormones during seed development of tartary buckwheat

	Number of DEGs	Biosynthesis	Metabolism	Receptor	Response	Signal transduction	Transportion
ABA	35	0	8	16	0	11	0
IAA	185	0	0	6	0	157	22
ZT	20	13	7	0	0	0	0
GA_3_	37	20	0	4	3	10	0
Total	277	33	15	26	3	178	22

### Expression patterns of DEGs related to flavonoids during seed development

Tartary buckwheat is rich in flavonoids, including rutin and other flavonoids [15]. As a result, 47 genes were annotated as related to flavonoids. Among these genes, the DEGs included 3 phenylalanine ammonia-lyase (PAL), 4 chalcone synthase (CHS), 2 chalcone isomerase (CHI), 8 flavonol synthase (FLS), 6 flavonoid 3',5'-hydroxylase, 14 UDP-glucose flavonoid, and 10 flavonoid 3'-monooxygenase (Additional file 1: Table S5). According to their level of expression by developmental stage, they have at least one highly expressed member at all developmental stages. In 13 DPA vs 19 DPA, the DEGs were mostly enriched in phenylalanine metabolism pathways (ath00360) in the KEGG database (FDR < 0.05) (Additional file 1: Figure S1b), including 1 DEG (Table 2). In 19 DPA vs 25 DPA, 4 DEGs were mostly enriched in flavonoid biosynthesis pathways (ath00941) in the KEGG database (FDR < 0.05) (Additional file 1: Figure S1c, Table 2). These DEGs showed a higher expression in the flavonoid biosynthesis pathway from 13 DPA to 19 DPA (Additional file 1: Figure S1a).

**Table 2:** Differentially expressed genes related to in flavonoid biosynthesis pathways during seed development of tartary buckwheat

Gene id	Related	Function category	Family	Stage	Readcount	Readcount	Log^2^FoldChange	Padj	Significant
FtPinG0008236900.01	PAL	Phenylpropanoid metabolism	PAL/histidase family	X2 vs X3	2444.836885	6450.386734	-1.3996	0.00817	DOWN
FtPinG0006907000.01	FLS	Flavonoid biosynthesis	iron/ascorbate-dependent oxidoreductase family	X3 vs X4	530.8399528	81.48645982	2.7036	0.024921	UP
FtPinG0008131000.01	CHS	Flavonoid biosynthesis	chalcone/stilbene synthases family	X3 vs X4	8286.397285	871.1066818	3.2498	0.00007048	UP
FtPinG0006907100.01	FLS	Flavonoid biosynthesis	iron/ascorbate-dependent oxidoreductase family	X3 vs X4	8600.271627	2007.552066	2.0989	1.5955E-06	UP
FtPinG0002790600.01	CHI	Flavonoid biosynthesis	chalcone isomerase family	X3 vs X4	1641.630481	287.3719632	2.5141	0.00007764	UP

### Expression patterns of DEGs related to starch during seed development

Starch is among the main nutrients in tartary buckwheat and dominates with relatively high proportions [16]. We identified 20 genes related to starch, including 5 soluble starch synthase, 9 starch synthase, and 6 granule-bound starch synthase genes, all of which belong to the glycosyltransferase 1 family (Table 3). In 13 DPA vs 19 DPA, no genes were differentially expressed (FDR < 0.05). In 19 DPA vs 25 DPA, 3 DEGs were involved in starch biosynthesis (Additional file 1: Figure S2).

**Table 3:** Genes related to starch synthesis (or metabolism) during seed development

Gene id	Related	Function category	Family	X2_fpkm	X3_fpkm	X4_fpkm
FtPinG0002952000.01	Starch synthase	Starch biosynthesis	glycosyltransferase 1 family	0	0.04209211	0
FtPinG0001526900.01	Starch synthase	Starch biosynthesis	glycosyltransferase 1 family	0.086131576	0	0
FtPinG0005431900.01	Starch synthase	Starch biosynthesis	glycosyltransferase 1 family	0	0	0
FtPinG0000359400.01	Starch synthase	Starch biosynthesis	glycosyltransferase 1 family	57.63315519	328.8316391	5.916537825
FtPinG0007470100.01	Starch synthase	Starch biosynthesis	glycosyltransferase 1 family	13.26934373	7.27664449	1.747617671
FtPinG0009838000.01	Starch synthase	Starch biosynthesis	glycosyltransferase 1 family	451.7534424	367.1546165	1.968141961
FtPinG0006332800.01	Starch synthase	Starch biosynthesis	glycosyltransferase 1 family	20.95952758	7.358124306	9.756228183
FtPinG0008723400.01	Starch synthase	Starch biosynthesis	glycosyltransferase 1 family	12.73350096	8.974233732	13.93766394
FtPinG0007775600.01	Starch synthase	Starch biosynthesis	glycosyltransferase 1 family	42.32919719	32.13196216	36.80551599
FtPinG0000021600.01	Starch synthase	Starch biosynthesis	glycosyltransferase 1 family	1.21734049	1.02004409	1.121365717
FtPinG0000380300.01	Starch synthase	Starch biosynthesis	glycosyltransferase 1 family	43.88186662	18.86806009	7.382532431
FtPinG0005565800.01	Starch synthase	Starch biosynthesis	glycosyltransferase 1 family	198.3185567	88.78020142	53.22576629
FtPinG0003226800.01	Starch synthase	Starch biosynthesis	glycosyltransferase 1 family	43.42372433	16.80944338	2.358411337
FtPinG0005368600.01	Starch synthase	Starch biosynthesis	glycosyltransferase 1 family	19.42316605	5.279935074	1.734551104
FtPinG0007534500.01	Starch synthase	Starch biosynthesis	glycosyltransferase 1 family	36.91435118	17.93308621	14.83261806
FtPinG0007419600.01	Starch synthase	Starch biosynthesis	glycosyltransferase 1 family	11.0790269	7.395763029	10.50993485
FtPinG0003227000.01	Starch synthase	Starch biosynthesis	glycosyltransferase 1 family	6.54592268	2.517975445	0.525192882
FtPinG0005939600.01	Starch synthase	Starch biosynthesis	glycosyltransferase 1 family	82.30668491	70.82139489	55.11481588
FtPinG0003590400.01	Starch synthase	Starch biosynthesis	glycosyltransferase 1 family	16.54472332	9.19667544	7.289004226
FtPinG0005109900.01	Starch synthase	Starch biosynthesis	glycosyltransferase 1 family	47.48674066	23.91252077	4.300813932

### Expression patterns of DEGs related to storage proteins during seed development

On the basis of differential solubility, storage proteins include globulin, albumin, glutelin, and prolamin [17]. As such, of the 21 genes, 18 are DEGs related to storage proteins, including 16 related to globulin and 2 related to glutelin (Additional file 1: Figure S3a). In 13 DPA vs 19 DPA, a GO analysis showed that the DEGs were significantly enriched in the molecular function term “nutrient reservoir activity” (GO:0045735) (Additional file 1: Figure S3b, c). In 19 DPA vs 25 DPA, the GO analysis showed that the DEGs were significantly enriched in the molecular function terms including nutrient reservoir activity (GO:0045735) and acireductone dioxygenase [iron(II)-requiring] activity (GO:0010309) (Additional file 1: Figure S3d, e).

## Discussion

### Correlation analysis between embryo cell enlargement and phytohormones during the development of buckwheat seed

**Fig. 8:**
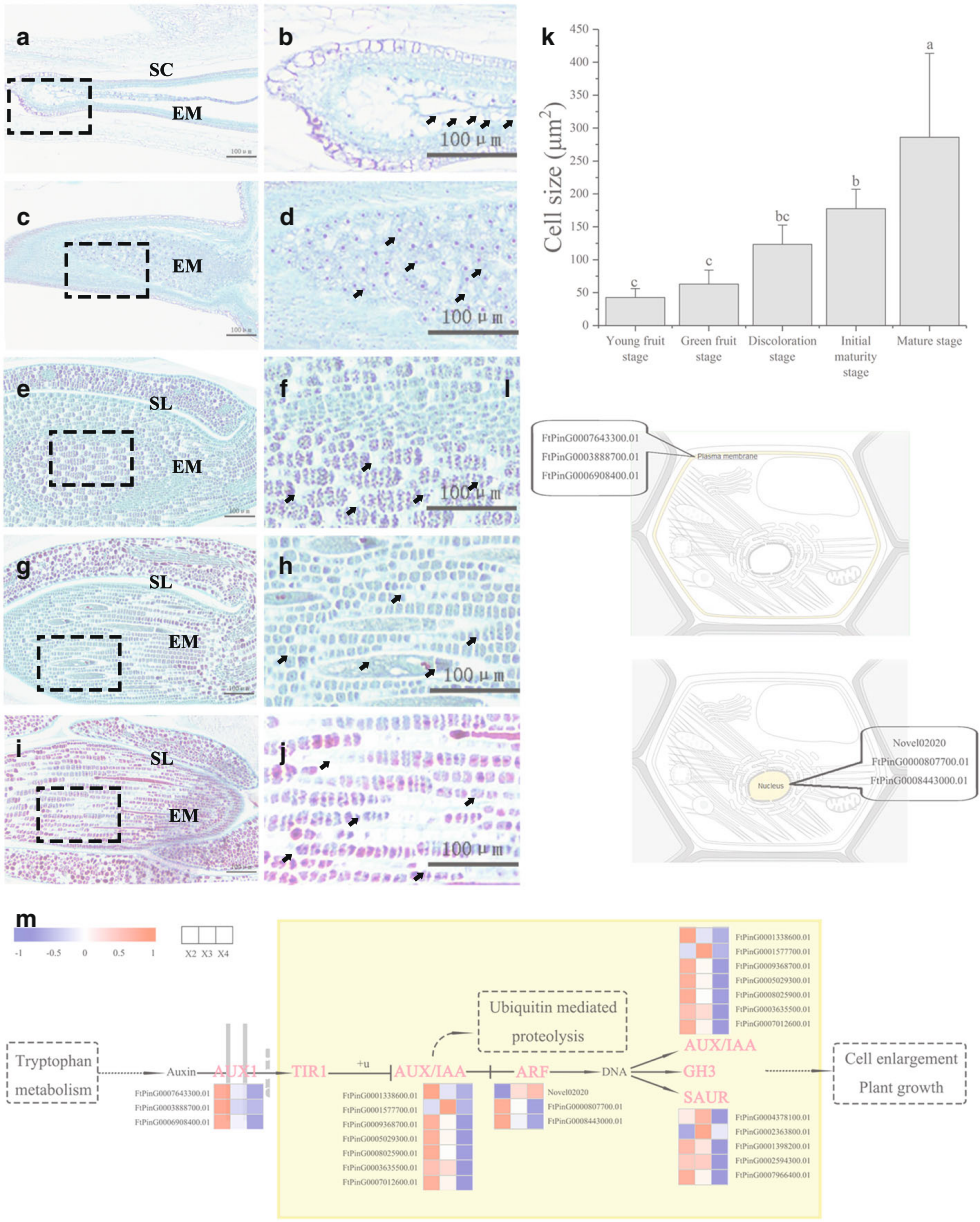
Enlargement of embryo cell during tartary buckwheat seed development. **a**, **c**, **e**, **g** and **i** Microscopic longitudinal sections of Tartary buckwheat seeds at 7, 13, 19, 25 and 30 DPA, respectively. **b**, **d**, **f**, **h** and **j** are the enlarged view of the boxes in (**a**), (**c**), (**e**), (**g**) and (**i**), respectively. SC (Seed cover). EM (Embryo). SL (Seminal leaf). The arrow represents the cell being measured. Bars = 100μm. **(k)** Embryo cell size at 7, 13, 19, 25 and 30 DPA. Error bars were obtained from five measurements. Small letter(s) above the bars indicate significant differences (α = 0.05, LSD) among treatments. **(l)** Subcellular location of DEGs related to embryo cell enlargement. This section provides information on the location in the cell (Graphics by Christian Stolte). **m** A simplified representation of the plant hormone signal transduction pathway of cell enlargement (adopted from the KEGG PATHWAY Database: http://www.genome.jp/kegg/pathway.html) shows the following: auxin influx carrier (AUX1), transport inhibitor response 1 (TIR1), auxin-responsive protein IAA (AUX/IAA), auxin response factor (ARF), auxin responsive GH3 gene family (CH3), and SAUR family protein (SAUR). The expression value of each gene is colored in log_10_(FPKM) in three samples X2 (13 DPA), X3 (19 DPA), and X4 (25 DPA)

The role of auxin in cell enlargement and the development of plant organs have been fully confirmed [18, 19], and the seed is no exception. In our study, the content of IAA decreased gradually as a fluctuation curve during the development of buckwheat seed (Fig. 2b), and we identified 18 DEGs in transcriptome; these DEGs were enriched in the plant hormone signal transduction pathway (ath04075) and may be related to cell enlargement (Fig. 8m). Most of these DEGs were downregulated from 13 DPA to 25 DPA. FtPinG0007643300.01, FtPinG0003888700.01, and FtPinG0006908400.01 were matched to the amino acid/polyamine transporter 2 family, whose members are located on the plasma membrane (Fig. 8l) and are involved in proton-driven auxin influx. In addition, 7 DEGs were matched to the Aux/IAA family and located in the nucleus. Aux/IAA proteins are short-lived transcription factors and act as inhibitors of early auxin response genes at low concentrations of auxin [20]. Novel02020, FtPinG0000807700.01, and FtPinG0008443000.01 were matched to the ARF family and are located in the nucleus (Fig. 8l). Auxin response factors (ARFs) are transcription factors that specifically bind to the DNA sequence 5'-TGTCTC-3' found in auxin-responsive promoter elements (AuxREs) [21–23]. Five DEGs were matched to the ARG7 family whose members are located in the nucleus, and these DEGs may play a role in apical hook development [24]. According to our previous research results and related research, during the development of buckwheat seeds we hypothesize that the extent of seed embryo cell enlargement gradually decreased, and the final embryo cell size was no longer increased and tended to stabilize. To test this hypothesis, we compared the size of seed embryo cells from 7 DPA to 30 DPA. However, the extent of seed embryo cell enlargement gradually increased (Fig. 8k). In fact, the decrease in auxin concentration may not be enough to stop cell enlargement. Auxin plays a role in initiation or acts as a ‘on’ switch via secondary regulators. Only lowering the auxin concentration (that is, removing the ‘on’ switch) does not reduce other phytohormones. Considering this hypothesis, a second ‘on’ switch may be required to accurately determine the time frame for cell enlargement [25–30]. It was found that the content of ABA is different in embryogenic cell clusters, globular embryos, torpedo embryos, and cotyledon embryos [31]. During embryonic development, ABA affects starch biosynthesis and carbohydrate absorption and directly regulates or initiates the biosynthesis of protein [32], DNA, and mRNA [33], all of which have an effect on the enlargement of embryonic cells [34]. According to these reports, embryonic cells continuously biosynthesize and absorb nutrients, resulting in an enlarged size under the influence of ABA. This fact may reinforce the idea of ABA as the second switch for cell enlargement. Therefore, we speculate that the increase in and prolongation of ABA content from 7 DPA to 30 DPA may be related to the enlargement of seed embryo cells. In the end, via the content of ABA, we confirmed that the content increased during the development of buckwheat seeds, which is consistent with the increase in seed embryo cells (Fig. 8k).

### Correlation analysis between dormancy and phytohormones during the development of buckwheat seed

**Fig. 9:**
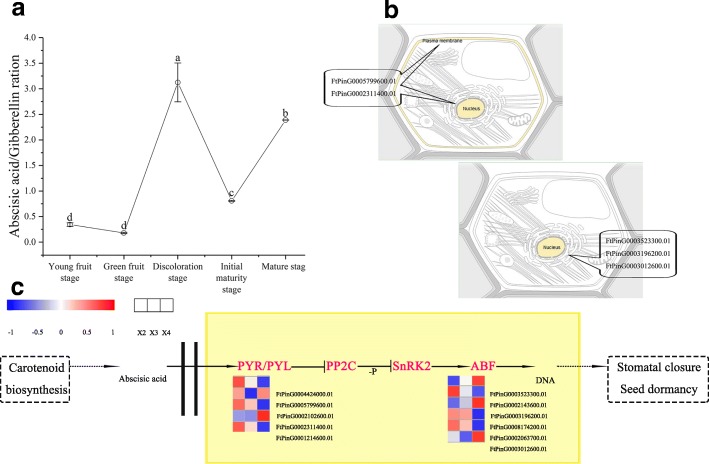
DEGs involved in the plant hormone signal transduction pathway of seed dormancy. **a** Corresponding ratios of ABA to GA_3_ at different development stages of Tartary buckwheat seed. **b** Subcellular location of DEGs related to seed dormancy. This section provides information on the location in the cell (Graphics by Christian Stolte). **c** A simplified representation of the plant hormone signal transduction pathway of seed dormancy (adopted from the KEGG PATHWAY Database: http://www.genome.jp/kegg/pathway.html) shows the following: abscisic acid receptor PYR/PYL family (PYR/PYL), protein phosphatase 2C (PP2C), serine/threonine-protein kinase SRK2 (SnRK2), and ABA responsive element binding factor (ABF). The expression value of each gene is colored in log_10_(FPKM) in three samples X2 (13 DPA), X3 (19 DPA), and X4 (25 DPA)

Dormancy occurs during seed development, and seeds are scattered from the parent plant in a dormant state [35]. ABA is a positive regulator of seed dormancy induction. Transgenic tobacco plants expressing anti-ABA antibody lack ABA, and the seeds are not dormant [36]. Overexpression of ABA biosynthesis genes can increase the content of ABA in seeds, thus promoting seed dormancy or delayed germination [37]. The biosynthesis of GA does not correlate with the establishment of primary dormancy [38]. However, GA_3_ plays important roles in the seed development of tomato, pea (*Pisum sativum*) and some *Brassica* plants, including fertilization, assimilation, embryonic growth, prevention of seed abortion, and fruit growth [39, 40]. A model of seed dormancy and germination was regulated by ABA and GA_3_, and the complex phenomenon was explained by the response to environmental factors. The model was affected not only by many genes but also by plant hormones and environmental factors [41, 42]. According to this model, the biosynthesis of ABA and the signal of GA_3_ catabolism determine the dormancy of the seed, and the biosynthesis of GA_3_ and the signal of ABA catabolism determine the transformation of seeds to begin germinating. Our data show that the content of ABA was significantly related to seed development (Fig. 4). The content of ABA increased with the development of buckwheat seeds, especially during 13 DPA to 25 DPA (Fig. 2d), when a large amount of storage material accumulated (Fig. 3). Moreover, only 2 DEGs (FtPinG0002387400.01 and FtPinG0003614600.01) that were involved in the oxidative degradation of ABA were downregulated during 19 DPA to 25 DPA (Fig. 7a). We also found the peak ABA/GA ratio occurred at 19 DPA (Fig. 9a). Based on the results we described earlier and those of related studies, we hypothesize that a large number of genes related to buckwheat seed dormancy are expressed from 19 DPA to 25 DPA. To test this hypothesis, we identified 11 transcriptome DEGs that were enriched in plant hormone signal transduction pathways (ath04075) and that were related to seed dormancy from 19 DPA to 25 DPA (Fig. 9c). Among them, the upregulated FtPinG0005799600.01 and FtPinG0002311400.01 belong to the PYR/PYL/RCAR abscisic acid intracellular receptor family, whose members are located on the plasma membrane and in the nucleus [43] (Fig. 9b). FtPinG0005799600.01 was matched to the abscisic acid receptor (PYL4) gene, and FtPinG0002311400.01 was matched to the abscisic acid receptor (PYL10) gene; both are ABA receptors required for ABA-mediated responses, and when activated by ABA, they inhibit the activity of group-A protein type 2C phosphatases (PP2Cs) [44–46]. In addition, the upregulated FtPinG0003523300.01, FtPinG0003196200.01, and FtPinG0003012600.01 belong to the bZIP family, whose members are located in the nucleus (Fig. 9c). FtPinG0003523300.01 was matched to the protein ABSCISIC ACID-INSENSITIVE 5, which participates in the regulation of ABA-regulated gene expression during seed development and joins the embryonic normative element and the ABA-responsive element (ABRE) of the Dc3 gene promoter and the ABRE gene promoter in the Em1 and Em6 genes [47–53]. FtPinG0003196200.01 and FtPinG0003012600.01 were matched to ABSCISIC ACID-INSENSITIVE 5-like protein 5, which is involved in the ABA response, acts as a positive component of glucose signal transduction, and specifically binds to the ABRE of the rd29B gene promoter [53–56]. As expected, a large number of genes related to buckwheat seed primary dormancy were expressed during 19 DPA to 25 DPA, and we identified good candidate genes related to buckwheat seed dormancy.

### Insights into the correlations between nutrient changes in and seed development

**Fig. 10:**
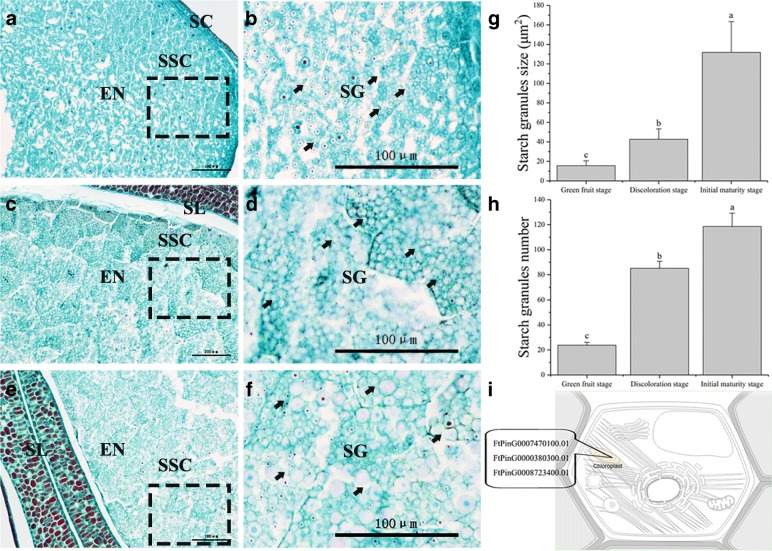
Comparison of starch granules morphology during different development stages of tartary buckwheat seed. **a**, **c**, and **e** Microscopic longitudinal sections of Tartary buckwheat seeds at 13, 19, and 25 DPA, respectively. **b**, **d**, and **f** are the enlarged view of the boxes in (**a**), (**c**), and (**e**), respectively. SC (Seed cover). EN (Endosperm). SL (Seminal leaf). SSC (Starch storage cells). SG (Starch granules). The arrow represents the cell being measured. Bars = 100μm. **g** Starch granules size at 13 DPA, 19 DPA, and 25 DPA. **h** Starch granules number (Average of total starch granules in each starch storage cell) at 13 DPA, 19 DPA, and 25 DPA. Error bars were obtained from five measurements for (**g**) and (**h**). Small letter(s) above the bars indicate significant differences (α = 0.05, LSD) among treatments. **i** Subcellular location of DEGs related to starch biosynthesis. This section provides information on the location in the cell (Graphics by Christian Stolte)

Starch is the major component of buckwheat nutrients and accounts for more than 86% of nutrients (Fig. 3e). Starch causes seeds and other storage organs to expand and enlarge during seed development (Fig. 1a); thus, it plays an important role in the size, weight and quality of buckwheat seeds during their development. In this study, starch was highly associated with seed size (especially the longitudinal diameter) during buckwheat seed development (Fig. 4). The seeds increased significantly during 7 DPA to 13 DPA (Figs 1b, 3b), which means buckwheat seeds and other storage organs expand and enlarge mainly during prophase (Fig. 1b). In our study, we identified 3 DEGs related to starch during seed development (Additional file 1: Figure S2). Among them, FtPinG0008723400.01 was matched to the SS4 gene, which is located in the chloroplast (Fig. 10i). SS4 may be involved in the initiation of starch granule formation and may play a regulatory role in controlling starch accumulation in plastids [57–59]. In our data, the expression of FtPinG0008723400.01 was upregulated only during 19 DPA to 25 DPA, and it is plausible to infer that a large number of starch granules were formed at the same time. To test this hypothesis, we compared the starch granule numbers and size at 13 DPA, 19 DPA and 25 DPA (Fig. 10). As expected, there was noticeable difference in starch granules numbers and size from 19 DPA to 25 DPA (Fig. 10g, h), suggesting that FtPinG0008723400.01 may be involved in the increase in starch granules and may play a regulatory role in controlling starch accumulation in starch storage cells. In addition, the expansion of starch storage cells was mainly due to the increase in starch granules size, not the increase in the number of starch granules (Fig. 10g, h). Furthermore, we found that FtPinG0007470100.01 and FtPinG0000380300.01 were matched to the WAXY gene, which is located in the chloroplast (Fig. 10i). This protein is involved in the biosynthesis of starch and is a part of glycan biosynthesis, which is responsible for the synthesis of the amylose component of starch. In our study, the expression of these genes was downregulated, and starch also decreased significantly from 19 DPA to 25 DPA (Fig. 3b). Hence, FtPinG0008723400.01, FtPinG0007470100.01 and FtPinG0000380300.01 are good candidate genes related to buckwheat starch biosynthesis during seed development.

Total protein, including proenzymes, enzymes, and metabolic regulatory substances, is the second most dominant component of buckwheat nutrients, and storage protein accounts for 2~6% of nutrients (Fig. 3e). During seed development, many proteins are regulatory factors that are involved in metabolism [60, 61], so protein is the most active part of buckwheat nutrients. In our data, the total protein content was strongly associated with seed development (Fig. 4). The total protein content increased significantly from 7 DPA to 25 DPA but then started to decrease gradually during 25 DPA to 30 DPA (Fig. 3c). The diversity of proteins increased with seed development, especially from 19 DPA to 25 DPA. It is plausible to infer that a large number of genes related to storage protein biosynthesis are expressed during 19 DPA to 25 DPA. To test this hypothesis, we identified 16 DEGs related to storage proteins (Additional file 1: Figure S3a) in the transcriptome. The expression of FtPinG0000417000.01 and FtPinG0008579200.01, which are related to glutelin, was upregulated during 19 DPA to 25 DPA (Additional file 1: Figure S3a); these genes were enriched in the molecular function terms nutrient reservoir activity (GO:0045735) and acireductone dioxygenase [iron (II)-requiring] activity (GO:0010309) (Additional file 1: Figure S3a). Glutelin is responsible for some refined baking properties in bread. High-molecular-weight (HMW) and low-molecular-weight (LMW) glutelins are commonly present in these substances. A HMW glutelin of the grass tribe *Triticeae* can be used as an intraperitoneal disease sensitizer for individuals with the HLA-DQ8 class II antigen receptor gene [62]. Hence, FtPinG0000417000.01 and FtPinG0008579200.01 were good candidate genes related to glutelin biosynthesis during seed development. Storage globulins of 11–12S in the starchy endosperm are also present in some grains [63]. Our data showed that the DEGs encoding the 13S globulin seed storage protein and the 11S globulin seed storage protein were upregulated mainly from 13 DPA to 19 DPA (Additional file 1: Figure S3a) and belong to the 11S seed storage protein (globulin) family. From a seed development standpoint, starchy endosperm is gradually absorbed by cotyledons during buckwheat seed development (Fig. 1a), providing nitrogen sources for cotyledon development during premetaphase in buckwheat seed development. Those DEGs were enriched in the molecular function term nutrient reservoir activity (GO:0045735) (Additional file 1: Figure S3a). The expression of only FtPinG0004288700.01 was upregulated from 19 DPA to 25 DPA (Additional file 1: Figure S3a). As expected, a large number of genes related to storage protein biosynthesis were expressed from 19 DPA to 25 DPA, and we identified strong candidate genes related to the biosynthesis of these storage proteins.

**Fig. 11:**
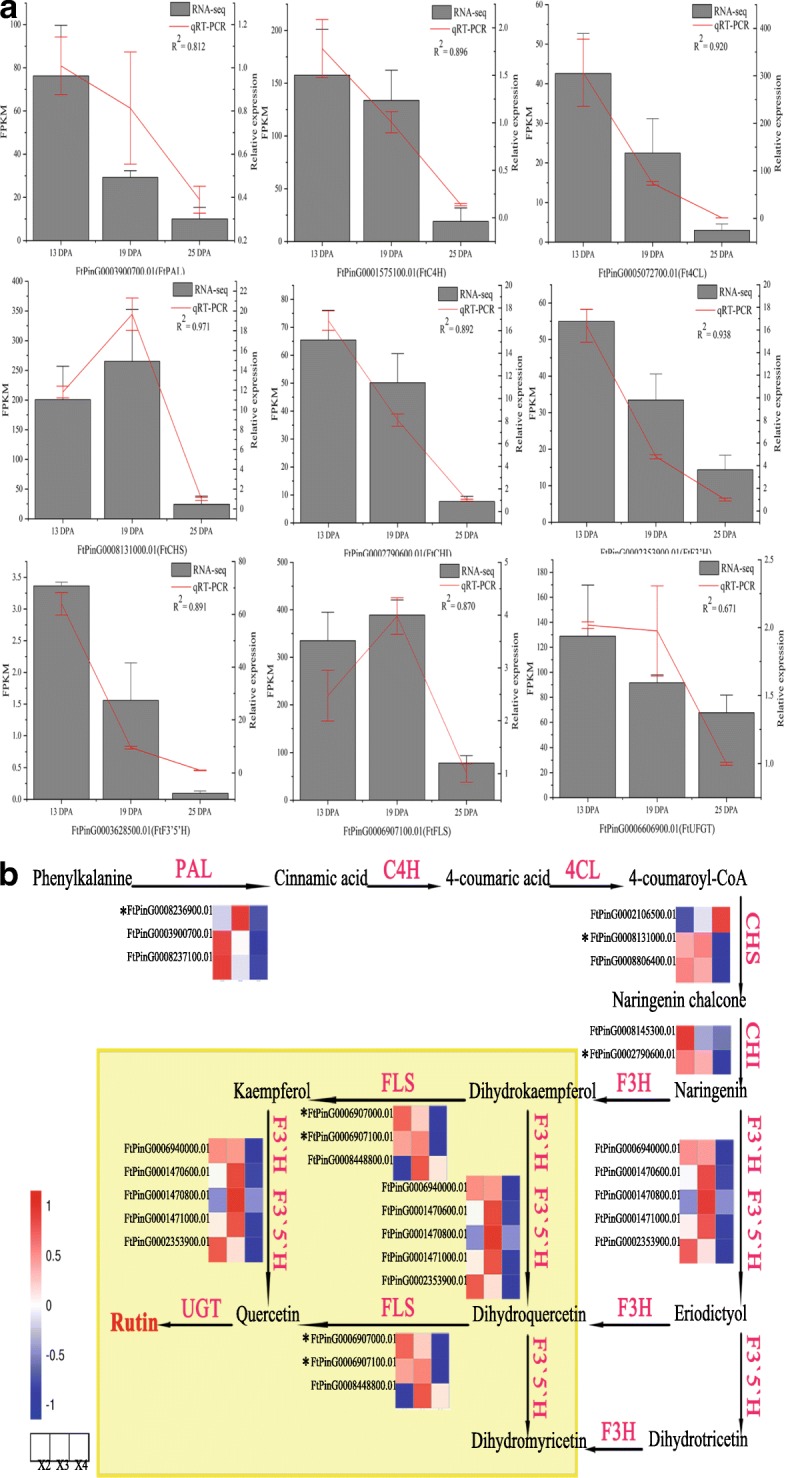
DEGs involved in rutin biosynthesis pathway. **a** RT- qPCR confirmation of 9 rutin biosynthesis related genes. **b** A simplified representation of the flavonoid biosynthetic pathway (adopted from the KEGG PATHWAY Database: http://www.genome.jp/kegg/pathway.html) shows the following enzymes: phenylalanine ammonia-lyase (PAL), cinnamate-4-hydroxylase (C4H), 4-coumarate Co A ligase (4CL), chalcone synthase (CHS), chalcone isomerase (CHI), flavanone-3’-hydroxylase (F3’H), flavanone-3-hydroxylase (F3H), flavonol synthase (FLS), glucosyl/rhamnosyl transferase, and flavanone-3’-5’-hydroxylase (F3’5’H). The expression value of each gene is colored in log_10_(FPKM) in three samples X2 (13 DPA), X3 (19 DPA), and X4 (25 DPA). * indicate the gene was differentially expressed during 13 DPA to 25DPA

Tartary buckwheat comprises a large group of widely grown medicinal and edible crops and is considered the main dietary source of rich rutin [15]. In mature buckwheat seeds, rutin accounts for 10% of the nutrients (Fig. 3e) and is an important secondary metabolite that reduces UV light damage as a key UV-B-absorbing compound [64]. Thus, rutin plays an important role in the development of buckwheat seed. In this study, the content of rutin increased gradually with the development of buckwheat seeds, especially during 25 DPA to 30 DPA (Fig. 3a). Among the genes related to rutin biosynthesis, we identified 5 DEGs from 13 DPA to 25 DPA (Additional file 1: Figure S1a). In addition, the expression patterns of 9 important rutin biosynthesis related genes were verified by RT-qPCR (Fig. 11a). In Fig. 11b, most of these DEGs in the upstream portion of the rutin biosynthesis pathway exhibited higher expression during 13 DPA to 19 DPA. Among them, FtPinG0008236900.01 was matched to the PAL gene. PAL is a key enzyme in plant metabolism and catalyzes the first step of the biosynthesis of phenylpropane skeletons by L-phenylalanine, leading to a variety of natural products. FtPinG0008131000.01 was matched to the CHS gene. The CHS protein is involved in the biosynthesis of flavonoids and plays in a role in the biosynthesis of secondary metabolites. The primary product of this enzyme is 4,2',4',6'-tetrahydroxychalcone, which can then spontaneously isomerize to naringin under certain conditions. FtPinG0002790600.01 was matched to the CHI gene; the encoded protein catalyzes the intramolecular cyclization of bicyclic chalcone to tricyclic (S)-flavanones and is responsible for the isomerization of 4,2',4',6'-tetrahydroxychalcone to naringenin. FtPinG0006907000.01 and FtPinG0006907100.01 were matched to the FLS gene; the FLS protein reacts with dihydroflavonol to produce flavonol, which can react with dihydrokaempferol to produce kaempferol. In turn, kaempferol can lead to the production of quercetin from dihydroquercetin, and catalysis of dihydromyricetin can yield myricetin. The high content of rutin in tartary buckwheat seeds may be related to these DEGs. Overall, we have identified strong candidate genes related to rutin biosynthesis during the development of buckwheat seeds.

## Conclusions

In recent years, the gene regulatory network governing the physiological changes occurring during seed development have receive little attention. The development of tartary buckwheat seeds was characterized by light and electron microscopy. The accumulation of plant hormones and nutrients was measured by high-performance liquid chromatography, and the expression of key genes was analyzed with the support of the genome of tartary buckwheat. By analyzing the relationship between plant hormone changes and seed development, we studied the embryo cell expansion and primary dormancy of tartary buckwheat seeds. In addition, by analyzing the relationship between nutritional changes and seed development, we determined the gene regulatory network controlling the accumulation of rutin, starch, storage protein and soluble sugars. We have provided abundant genomic resources for tartary buckwheat and Polygonaceae communities and carried out novel molecular studies on the correlations between the physiological changes in and the seed development of tartary buckwheat.

## Methods

### Plant materials and phenotype investigations

Seeds of tartary buckwheat (Xiqiao No. 2) were collected in 2016 from the experimental field of the College of Life Science, Sichuan Agricultural University (Lat. 29°97’ N, 102°97’ E, Alt. 580 m), China. We observed the development of seeds from anthesis until maturation in April-May, 2016. Seeds were collected manually every two days from the onset of seed set until maturity (Fig. 1), covering a total span of 30 days, after which the size and weight of the developing seeds were measured. For phytohormone, rutin and nutrient sampling, seeds at five development stages (7, 13, 19, 25, and 30 DPA) were collected from the same individual. For the transcriptome sampling, seeds at three development stages (13, 19, and 25 DPA) were collected from the same individual. In addition, seeds at the same development stage were collected from three replicate plants. The samples were immediately flash frozen in liquid nitrogen and stored at −80°C for further use.

### Measurement of phytohormone contents

Fresh samples that were weighed to approximately 0.5 g were ground in liquid nitrogen. The powder was subsequently homogenized in 10 mL of 80% methanol, after which the solution was stirred overnight at 4°C. This suspension was then centrifuged at 12 000 rpm for 10 min under refrigeration (4°C). The supernatant was collected, after which 5 mL of 80% methanol was added to the residue. Similarly, the supernatant was collected after centrifugation. The pooled supernatant (~15 mL) was flash evaporated at 36°C until the methanol vaporized (~3 mL). A bottle was washed with 5 mL of ultrapure water, and then the water was combined with the residual liquid (~3 mL). The solution was decolorized with 15 mL of diethyl ether three times, after which the ether phase was discarded. The aqueous phase was collected and basified to pH 8.0 with 0.1 M Na_2_HPO_3_. The basified extract was kept in a shaker for 30 min with 50 mg of polyvinylpyrrolidone (PVP) at 4°C. In addition, the extract was centrifuged at 12 000 rpm for 10 min. The supernatant was subsequently collected and was acidified to pH 3.0 with 0.2 M citric acid. The resultant solution was partitioned three times against 5 mL of ethylacetate, after which the aqueous phase was discarded. The pooled ethylacetate phase (~15 mL) was flash evaporated at 36°C to near dryness. Afterward, the residue was dissolved in 1 mL of methanol [65–67].

The sample was filtered through a nylon 66 filter (25 mm diameter, 0.45 μm pore size) prior to injection into a high-performance liquid chromatograph. HPLC analysis was performed on an Agilent 1260 system using a C18-ODS (3.5 μm × 150 mm × 4.6 mm) column (Agilent, USA) and a UV/VIS detector. An injection volume of 10 μL, column temperature of 35°C, flow rate of 1 mL min^-1^, and run time of 10 min were maintained for all analyses. The system was calibrated with external standards of IAA, GA_3_, ABA, and ZT. For detection, separation was performed with a mixture of methanol and distilled water containing 0.6% acetic acid (V:V = 50:50) following isocratic elution. The elutant was scanned at 257 nm.

### Measurement of rutin contents

The seeds of tartary buckwheat were dried at 60°C for 4 h, milled into a fine powder, and filtered through a 0.177 mm sieve. A total of 0.10 g of dry powder for each tissue was mixed with 1 mL of methanol and incubated at 60°C using an ultrasonic bath for 2 h. The mixture was then centrifuged at 4 000 r min^-1^ for 10 min, after which the supernatant was collected. The supernatant’s pH value was adjusted to 6 using 0.1 mol L^-1^ phosphoric acid and then filtered through 0.22 μm membrane filter. High-performance liquid chromatography was performed on an Agilent 1260 system using an Agilent C18 column (4.6 mm × 150 mm, 5 μm) (Agilent Technologies, USA) at 30°C. The mobile phase consisted of methanol:0.5% phosphoric acid (V/V) = 40:60 at flow rate of 1 mL min^-1^ with a total injection volume of 10 μL. The rutin quantity was estimated based on the linear calibration curve of standard rutin hydrate (98%) (Yuanye, China) at a detection wavelength of 257 nm. Three independent sample analyses were performed for each tissue [68].

### Measurement of nutrient contents

The contents of total soluble sugars in buckwheat seeds at different developmental periods were measured by anthrone-H_2_SO_4_ colorimetry. Ground sample tissue (0.07 g) and 7 mL of distilled water were put in a 10 mL graduated test tube that was closed with a stopper. The mixture was then extracted in a water bath for 30 min at 100°C and centrifuged at 4 000 × g for 5 min, after which the supernatant was transferred to a 50 mL volumetric flask (extracted twice). Afterward, 100 μL of the extract was pipetted into the test tube, and 3 ml of anthrone reagent was added, after which the tube was placed in a boiling water bath for 10 min. The change in absorbance at 620 nm was then measured, after which the soluble sugar content was calculated according to the standard curve [69].

The total protein content was measured using the Coomassie brilliant blue G-250 method. A standard protein solution and a Coomassie brilliant blue G-250 solution were prepared, and fresh seeds (0.5 g) were ground in 10 mL of distilled water using a mortar and pestle. After the solution was centrifuged at 4000 × g for 10 min, the supernatant was transferred to a new tube to assay the total protein content, and the change in absorbance at 595 nm was measured [69].

The contents of starch in the seeds of buckwheat were measured by anthrone-H_2_SO_4_ colorimetry [70]. The solid residues that extract soluble sugars were used to test the starch content. First, 20 mL of hot water was added to the tube that contained solid residues. An extraction in a water bath (100°C) for 15 min then occurred, after which 2 mL of 9.2 mol L^−1^ perchloric acid was added; the water bath (100°C) extraction then continued for 15 min. After cooling and mixing the extract, the filtrate was brought to a constant volume in a 50 mL measuring flask. Afterward, 100 μL of the extract was pipetted into the test tube, and 3 mL of anthrone reagent was added. The extract was then placed in a boiling water bath for 10 min. The change in absorbance at 620 nm was then measured, and the starch content was calculated according to the standard curve [69].

### Statistical analysis

All the data were analyzed by analysis of variance using the Origin Pro 2017 statistics program, and the means were compared by the least significant difference test (LSD) at the 0.05 level of significance. The Pearson's correlation coefficient between DPA, rutin, starch, total proteins, soluble sugars, ZT, GA_3_, IAA, ABA, transverse diameter, longitudinal diameter, transverse diameter × longitudinal diameter, and seed weight were analyzed by Origin Pro 2017 software.

### Total RNA extraction, cDNA Library construction, and high-throughput sequencing

The total RNA samples were extracted using the TRIzol reagent (Invitrogen) and were then treated by RNase-free DNase I (Takara) to remove the genomic DNA. mRNA libraries were constructed according to the standard protocols provided by Illumina. The quality of mRNA, including purity, quantity and integrity, was tested using Nanodrop, Qubit, and Agilent 2100 systems. The mRNA was enriched using Dynabeads oligos (dTs) (Dynal; Invitrogen) and fragmented using fragmentation buffer. Double-stranded cDNAs were synthesized using both reverse transcriptase (Superscript II; Invitrogen) and random hexamer primers and were further purified using AMPure XP beads. Finally, the purified double-stranded cDNA samples were further enriched by PCR to construct the final cDNA libraries that were sequenced using a HiSeq 4000 (150 bp paired ends) by Novogene (China). All raw sequence read data were uploaded to the NCBI Sequence Read Archive (SRA, http://www.ncbi.nlm.nih.gov/Traces/sra) under accession number GSE111937.

### Map sequencing results and differential expression analysis

Adaptor sequences and low-quality sequences were removed from the raw reads (Q < 20). The clean reads were aligned to the reference genome sequences of the tartary buckwheat (Pinku1) genome (http://www.mbkbase.org/Pinku1/) using TopHat (v2.0.12). The default parameters were used, allowing mismatches of no more than two bases. A reference-based assembly of all the reads was performed using the Cufflinks v2.1.1 reference annotation-based transcript (RABT) assembly method. The assembled transcript fragments were compared with the reference annotation to predict new genes and novel exons and to optimize gene structures using Cuffcompare.

Gene expression differences in the different sample pairs were detected using the DESeq package (v1.10.1). In this study, to investigate the genes involved in tartary buckwheat seed development, the two sample pairs (13 DPA vs 19 DPA and 19 DPA vs 25 DPA) were used to investigate the differentially expressed genes under different conditions. The P-value threshold was determined using the FDR for multiple tests [71]. The thresholds were set using an FDR ≤ 0.05 and the absolute value of log_2_ (fold change) with FPKM ≥ 1 to determine significant differences in gene expression. The FPKM was used to eliminate the influence of different gene lengths and sequencing discrepancies on the quantification of gene expression to ensure direct comparison of gene expression between different sample pairs [72].

### Functional classification of differentially expressed genes

An analysis of functional enrichment, including GO, was performed to identify which DEGs were significantly enriched in GO terms. GO enrichment of the DEGs was conducted using the GOseq R package (release 2.12). GO terms with a corrected P-value < 0.05 were considered significantly enriched by the differentially expressed genes. The GO annotations were functionally classified using WEGO software for gene function distributions. KOBAS software (v2.0) was used to identify the statistical enrichment of the differentially expressed genes in the KEGG pathways. The pathways with an FDR value of less than 0.05 were considered those genes showing significantly differential expression.

### Real-Time PCR confirmation of differentially expressed genes

Quantitative real-time PCR analysis was performed to confirm transcriptome results. The corresponding sequences of these genes were obtained from the tartary buckwheat (Pinku1) genome sequence database. The RT-qPCR primers were designed according to the transcript sequences of 20 genes using Primer3 software (http://frodo.wi.mit.edu/) (Additional file 1: Table S7). The *FtH3* gene was used as the internal control. RT-qPCR experiments were replicated at least three times.

First-strand cDNA was synthesized from 1 mg of DNase I-treated RNA samples in a 40 μL reaction solution with random primers, using a PrimeScript RT Reagent Kit with gDNA Eraser (TaKaRa). Standard RT–qPCR was performed using SYBR Premix Ex Taq II (TaKaRa) on a CFX96 Real Time System (BioRad). Data were analyzed by the 2^−(∆∆Ct)^ method to obtain relative mRNA expression data [73].

## Additional file

Additional file 1: **Figure S1.** DEGs related to flavonoid in three samples. **Figure S2.** DEGs related to starch in three samples. **Figure S3.** DEGs related to storage protein in three samples. **Table S1.** Quality of the RNA sequencing data. **Table S2.** Information of reads aligned to the reference genome sequence. **Table S3.** Pearson correlation between RNA-seq data from different samples. **Table S4.** List of genes were related to phytohormones during seed development. **Table S5.** List of genes were related to flavonoid during seed development. **Table S6.** Validation of the transcriptome data by qRT-PCR. **Table S7.** Primers sequences. (PDF 817 kb)

## Abbreviations

ABA: Abscisic acid
ABRE: ABA-responsive element
AGPase: ADP-glucose pyrophosphorylase
ARFs: Auxin response factors
AuxREs: Auxin-responsive promoter elements
BE: Branching enzyme
CHI: Chalcone isomerase
CHS: Chalcone synthase
DBE: Starch debranching enzyme
DEGs: Differentially expressed genes
DPA: Days postanthesis
DW: Dry weight
FDR: False discovery rate
FLS: Flavonol synthase
FPKM: Fragments per kilobase of transcripts per million mapped fragments
FW: Fresh weight
GA_3_: Gibberellin
GBSS: Granule-bound starch synthase
GO: Gene Ontology
HMW: High-molecular-weight
HPLC: High efficiency liquid chromatography
IAA: Indole-3-acetic acid
LMW: Low-molecular-weight
LSD: Least significant difference test
PAL: Phenylalanine ammonia-lyase
PCA: Principal component analysis
PP2Cs: 2C phosphatases
PVP: Polyvinylpyrrolidone
PYL10: Abscisic acid receptor
PYL4: Abscisic acid receptor
RNA-seq: RNA sequencing
RT-qPCR: Real-time Quantitative polymerase chain reaction
SS: Soluble starch synthase
SUS: Sucrose synthase
UGPase: UDP-glucose pyrophosphorylase
ZT: Zeatin
