# Supplementary material for: Insights into the correlation between Physiological changes in and seed development of tartary buckwheat (Fagopyrum tataricum Gaertn.)
Source: BMC Genomics. 2018 Aug 31;19:648. doi: 10.1186/s12864-018-5036-8 (PMC6119279; doi:10.1186/s12864-018-5036-8)
Supplement: Supplementary file 1 — Figure S1. DEGs related to flavonoid in three samples. Figure S2. DEGs related to starch in three samples. Figure S3. DEGs related to storage protein in three samples. Table S1. Quality of the RNA sequencing data. Table S2. Information of reads aligned to the reference genome sequence. Table S3. Pearson correlation between RNA-seq data from different samples. Table S4. List of genes were related to phytohormones during seed development. Table S5. List of genes were related to flavonoid during seed development. Table S6. Validation of the transcriptome data by qRT-PCR. Table S7. Primers sequences. (PDF 817 kb) [file 12864_2018_5036_MOESM1_ESM.pdf]

Title: Insights into the Correlation between Physiological Changes in and Seed Development of Tartary Buckwheat (*Fagopyrum tataricum* Gaertn.)

Running Title: Tartary Buckwheat Seed Development

Moyang Liu†, Zhaotang Ma†, Tianrun Zheng, Wenjun Sun, Yanjun Zhang, Weiqiong Jin, Junyi Zhan, Yuntao Cai, Yujia Tang, Qi Wu, Zizhong Tang, Tongliang Bu, Chenglei Li and Hui Chen※

Sichuan Agricultural University, College of Life Science, Ya'an, China

†Contributed equally

※**Correspondence**: chenhui@sicau.edu.cn

Co-authors email address:

lmyyunxi@163.com, WaohUncle_Ma@163.com, kobezey@163.com, sunnan82475@163.com, 15227793373@163.com, Joan541573@163.com, zhanjunyi0412@163.com, caiyt410725@163.com, 2049528646@qq.com, wuqi@sicau.edu.cn, 3530279123456789@163.com, tlbu@163.com, gezhucao1998@yahoo.com.cn

Additional file Figures:

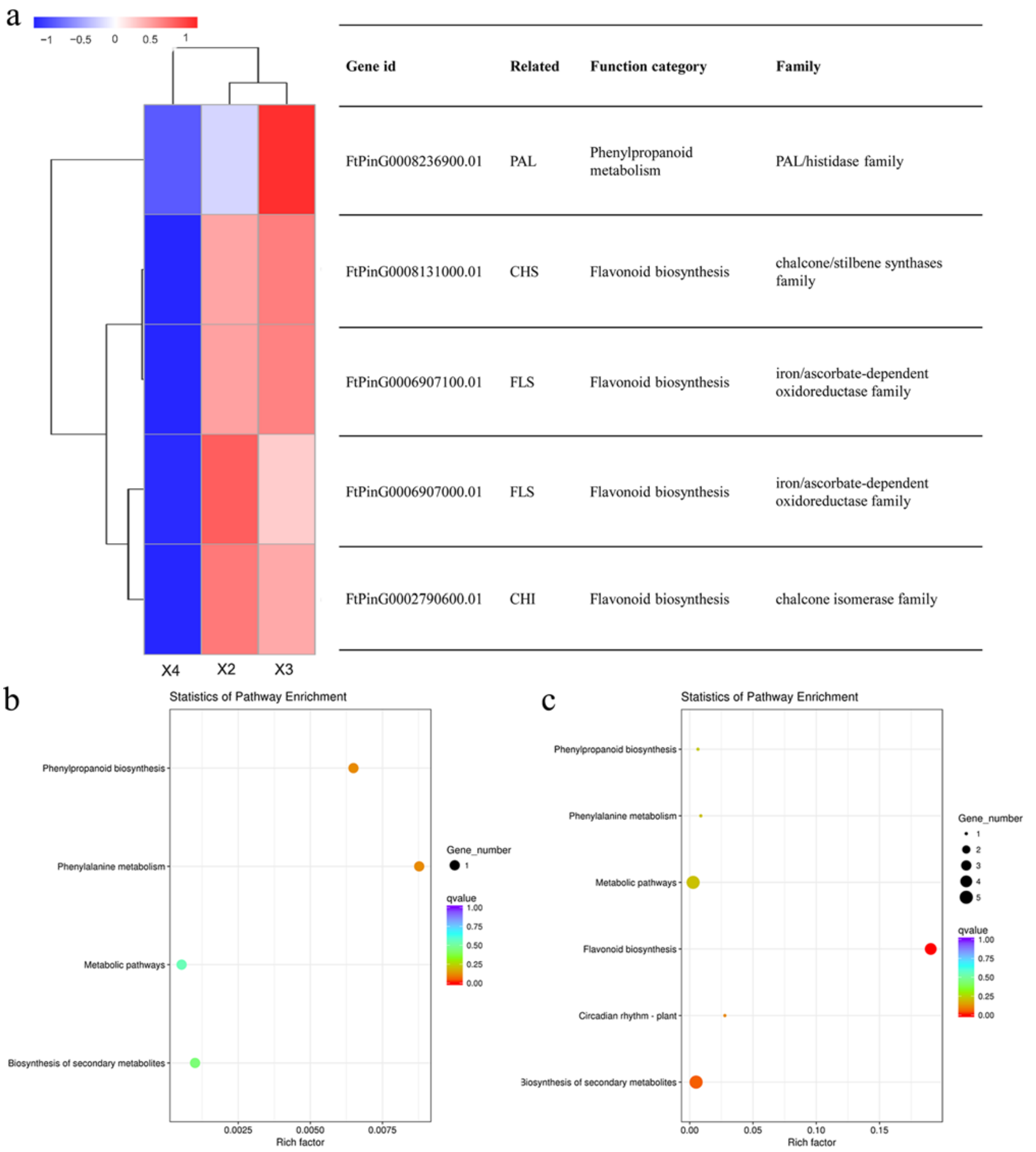

Gene id	Related	Function category	Family
FtPinG0008236900.01	PAL	Phenylpropanoid metabolism	PAL/histidase family
FtPinG0008131000.01	CHS	Flavonoid biosynthesis	chalcone/stilbene synthases family
FtPinG0006907100.01	FLS	Flavonoid biosynthesis	iron/ascorbate-dependent oxidoreductase family
FtPinG0006907000.01	FLS	Flavonoid biosynthesis	iron/ascorbate-dependent oxidoreductase family
FtPinG0002790600.01	CHI	Flavonoid biosynthesis	chalcone isomerase family

Figure S1 DEGs related to flavonoid in three samples.

(a) Hierarchical cluster of the DEGs related to flavonoid in X2 (13 DPA), X3 (19 DPA), and X4 (25 DPA). Red: high expression; Blue: low expression.

(b) Scatterplot of KEGG pathway enrichment in X2 (13 DPA) vs X3 (19 DPA) (FDR< 0.05).

(c) Scatterplot of KEGG pathway enrichment in X3 (19 DPA) vs X4 (25 DPA) (FDR< 0.05).

Rich factor is the ratio of the number of DEGs to the number of background genes in a KEGG pathway.

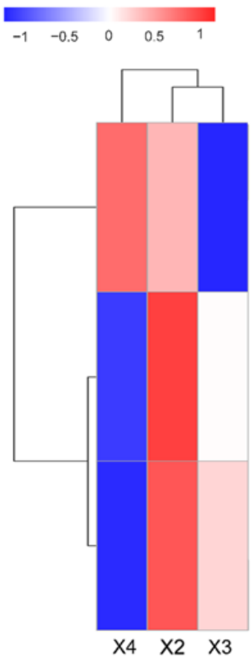

Gene id	Gene name	Related	Function category	Family
FtPinG0008723400.01	SS4	Starch synthase	Starch biosynthesis	glycosyltransferase 1 family
FtPinG0000380300.01	WAXY	Starch synthase	Starch biosynthesis	glycosyltransferase 1 family
FtPinG0007470100.01	WAXY	Starch synthase	Starch biosynthesis	glycosyltransferase 1 family

Figure S2 DEGs related to starch in three samples. Hierarchical cluster of the DEGs related to starch in X2 (13 DPA), X3 (19 DPA) and X4 (25 DPA). Red: high expression; Blue: low expression.

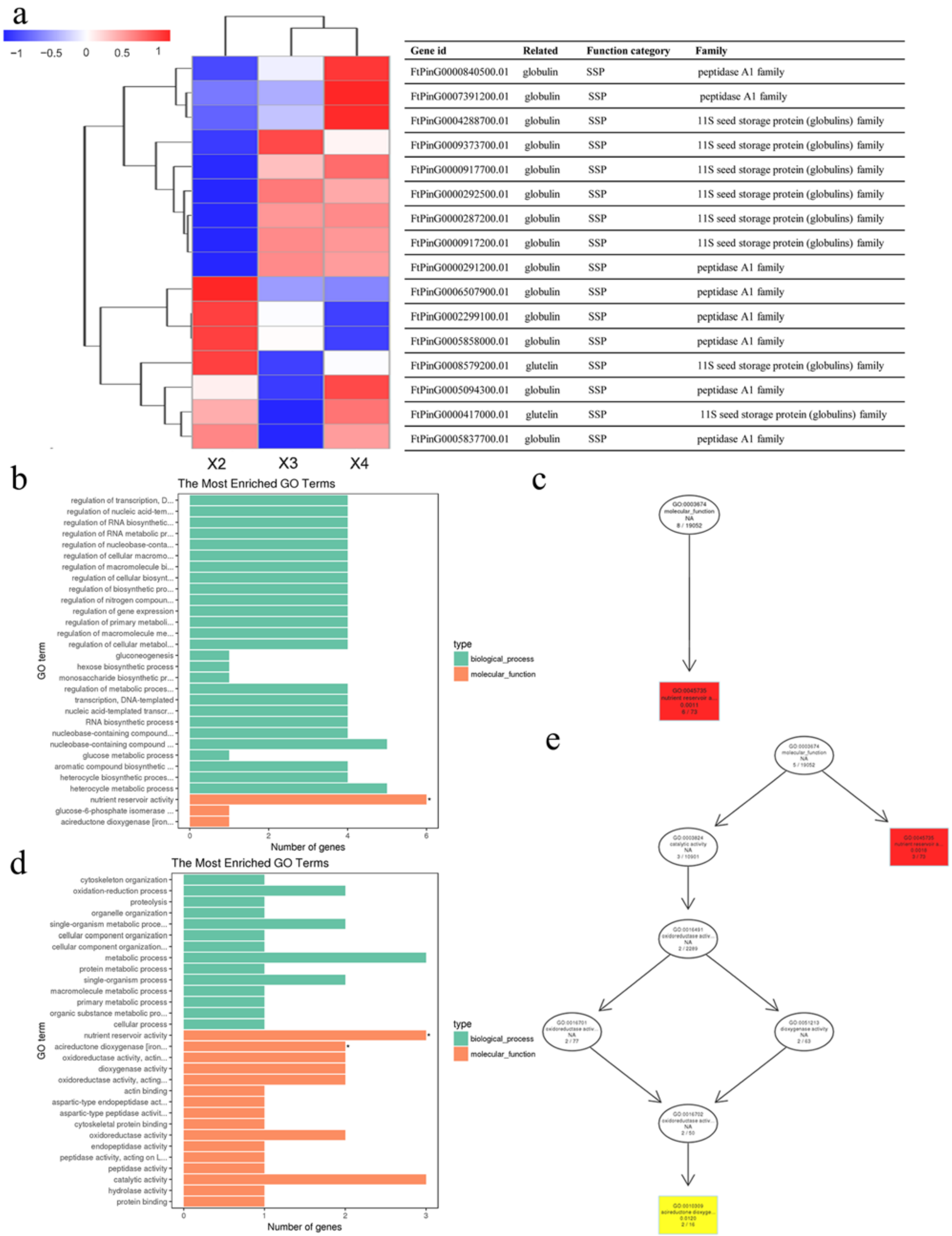

Gene id	Related	Function category	Family
FtPinG0000840500.01	globulin	SSP	peptidase A1 family
FtPinG0007391200.01	globulin	SSP	peptidase A1 family
FtPinG0004288700.01	globulin	SSP	11S seed storage protein (globulins) family
FtPinG0009373700.01	globulin	SSP	11S seed storage protein (globulins) family
FtPinG0000917700.01	globulin	SSP	11S seed storage protein (globulins) family
FtPinG0000292500.01	globulin	SSP	11S seed storage protein (globulins) family
FtPinG0000287200.01	globulin	SSP	11S seed storage protein (globulins) family
FtPinG0000917200.01	globulin	SSP	11S seed storage protein (globulins) family
FtPinG0000291200.01	globulin	SSP	peptidase A1 family
FtPinG0006507900.01	globulin	SSP	peptidase A1 family
FtPinG0002299100.01	globulin	SSP	peptidase A1 family
FtPinG0005858000.01	globulin	SSP	peptidase A1 family
FtPinG0008579200.01	glutelin	SSP	11S seed storage protein (globulins) family
FtPinG0005094300.01	globulin	SSP	peptidase A1 family
FtPinG0000417000.01	glutelin	SSP	11S seed storage protein (globulins) family
FtPinG0005837700.01	globulin	SSP	peptidase A1 family

Figure S3 DEGs related to storage protein in three samples.

(a) Hierarchical cluster of the DEGs related to storage protein in X2 (13 DPA), X3 (19 DPA), and X4 (25 DPA). Red: high expression; Blue: low expression.

(b) GO classification of DEGs in X2 (13 DPA) vs X3 (19 DPA) (FDR< 0.05). The top 30 enriched

GO classifications are listed. Stars above bars indicate the amounts of differentially expressed genes are significantly higher or lower than the amounts of genes in random samples from the GO classification of all genes.

(c) The DEGs related to storage protein enriched molecular function in X2 (13 DPA) vs X3 (19 DPA). The different color frames indicate the extent of significance. Red: extremely significant.

(d) GO classification of DEGs in X3 (19 DPA) vs X4 (25 DPA) (FDR< 0.05). The top 30 enriched GO classifications are listed. Stars above bars indicate the amounts of differentially expressed genes are significantly higher or lower than the amounts of genes in random samples from the GO classification of all genes.

(e) The DEGs related to storage protein enriched molecular function in X3 (19 DPA) vs X4 (25 DPA). The different color frames indicate the extent of significance. Yellow: significant; Red: extremely significant.

Additional file Tables:

Table S1 Quality of the RNA sequencing data

Sample name	Raw reads	Clean reads	Clean bases	Error rate (%)	Q20(%)	Q30(%)	GC content (%)
X2_1	57,667,990	55,636,848	8.35G	0.02	97.01	92.69	46.53
X2_2	63,296,504	60,693,148	9.1G	0.02	96.72	92.2	47.6
X2_3	51,869,420	50,731,030	7.61G	0.02	97.2	93.21	45.9
X3_1	58,114,304	56,067,612	8.41G	0.02	96.66	92.13	49.21
X3_2	60,541,944	57,761,526	8.66G	0.02	96.51	91.88	49.84
X3_3	56,100,570	54,824,078	8.22G	0.02	96.61	92.13	48.79
X4_1	59,984,306	57,627,070	8.64G	0.02	96.43	91.74	49.42
X4_2	60,858,178	58,529,782	8.78G	0.02	96.53	91.84	49.31
X4_3	60,420,760	58,925,604	8.84G	0.02	96.56	92.09	49.79

Table S2 Information of reads aligned to the reference sequence

Sample name	Total reads	Total mapped	Multiple mapped	Uniquely mapped	Non-splice reads	Splice reads
X2_1	55636848	51764090 (93.04%)	1930613 (3.47%)	49833477 (89.57%)	31748015 (57.06%)	18085462 (32.51%)
X2_2	60693148	56174531 (92.55%)	2525111 (4.16%)	53649420 (88.39%)	34896700 (57.5%)	18752720 (30.9%)
X2_3	50731030	46824320 (92.3%)	1497685 (2.95%)	45326635 (89.35%)	27836598 (54.87%)	17490037 (34.48%)
X3_1	56067612	51667347 (92.15%)	3901556 (6.96%)	47765791 (85.19%)	31741951 (56.61%)	16023840 (28.58%)
X3_2	57761526	52901817 (91.59%)	3919826 (6.79%)	48981991 (84.8%)	34020967 (58.9%)	14961024 (25.9%)
X3_3	54824078	50452533 (92.03%)	3954895 (7.21%)	46497638 (84.81%)	30343350 (55.35%)	16154288 (29.47%)
X4_1	57627070	52064699 (90.35%)	4342596 (7.54%)	47722103 (82.81%)	32780130 (56.88%)	14941973 (25.93%)
X4_2	58529782	53886743 (92.07%)	4236381 (7.24%)	49650362 (84.83%)	34002356 (58.09%)	15648006 (26.74%)
X4_3	58925604	53854623 (91.39%)	4186267 (7.1%)	49668356 (84.29%)	34557038 (58.65%)	15111318 (25.64%)

Table S3 Pearson correlation between RNA-seq data from different samples

R^2	X2_1	X2_2	X2_3	X3_1	X3_2	X3_3	X4_1	X4_2	X4_3
X2_1	1	0.945	0.955	0.898	0.807	0.874	0.641	0.698	0.652
X2_2	0.945	1	0.904	0.936	0.889	0.906	0.728	0.784	0.74
X2_3	0.955	0.904	1	0.849	0.757	0.873	0.622	0.673	0.639
X3_1	0.898	0.936	0.849	1	0.938	0.955	0.778	0.829	0.786
X3_2	0.807	0.889	0.757	0.938	1	0.904	0.838	0.887	0.845
X3_3	0.874	0.906	0.873	0.955	0.904	1	0.773	0.822	0.794
X4_1	0.641	0.728	0.622	0.778	0.838	0.773	1	0.961	0.958
X4_2	0.698	0.784	0.673	0.829	0.887	0.822	0.961	1	0.961
X4_3	0.652	0.74	0.639	0.786	0.845	0.794	0.958	0.961	1

Table S4 List of genes were related to phytohormones during seed development.

Gene ID	Related Hormone	Function category	Family	X2 fpkm	X3 fpkm	X4 fpkm
FtPinG0003501200.01	auxin	Hormone signal transduction	Aux/IAA family	10.87657171	5.591377013	6.132366513
FtPinG0009172300.01	auxin	Hormone signal transduction	ARG7 family	0.209598055	0.061033937	0.030453959
FtPinG0000418400.01	auxin	Hormone signal transduction	ARG7 family	0.221751047	0.172045728	0.099360845
FtPinG0009166400.01	auxin	Hormone signal transduction	ARG7 family	0.378330683	0	0
FtPinG0009170000.01	auxin	Hormone signal transduction	ARG7 family	0.828795767	0.249491265	0.029875198
FtPinG0009167500.01	auxin	Hormone signal transduction	ARG7 family	2.399935389	0.135630132	0
FtPinG0009168600.01	auxin	Hormone signal transduction	ARG7 family	0	0	0
FtPinG0009169600.01	auxin	Hormone signal transduction	ARG7 family	5.582575197	0.615299977	0
FtPinG0009170500.01	auxin	Hormone signal transduction	ARG7 family	0	0	0
FtPinG0006689400.01	gibberellin	Hormone biosynthesis	iron/ascorbate-dependent oxidoreductase family	3.980492887	4.331099735	0.060697809
FtPinG0006689500.01	gibberellin	Hormone biosynthesis	iron/ascorbate-dependent oxidoreductase family	8.563137844	10.93041837	1.240128394
FtPinG0000344600.01	gibberellin	Hormone biosynthesis	iron/ascorbate-dependent oxidoreductase family	0.057631895	0.015402791	0
FtPinG0004797700.01	auxin	Hormone signal transduction	ARG7 family	11.21869515	19.89318875	11.54381144
FtPinG0000507900.01	auxin	Hormone signal transduction	ARG7 family	4.045828868	1.329497853	1.71885659
FtPinG0000271800.01	auxin	Hormone signal transduction	ARG7 family	1.799019134	0.386658648	0.21841858
FtPinG0005900800.01	cytokinin	Hormone metabolism	oxygen-dependent FAD-linked oxidoreductase family	0.016855302	0	0.008944136
FtPinG0003227500.01	auxin	Hormone signal transduction	Aux/IAA family	12.65977487	11.48602243	6.408427354
FtPinG0007231100.01	auxin	Hormone signal transduction	Aux/IAA family	15.87680968	12.87033941	8.884561389
FtPinG0004315700.01	auxin	Hormone signal transduction	Aux/IAA family	149.8830172	81.37146889	44.67748771
FtPinG0009157200.01	auxin	Hormone signal transduction	Aux/IAA family	4.46971304	1.564672036	1.089589018
FtPinG0002846500.01	auxin	Hormone signal transduction	Aux/IAA family	0.407381033	0.388560565	0.15959321
FtPinG0006568700.01	auxin	Hormone signal transduction	Aux/IAA family	0.780242565	0.018132196	0
FtPinG0007012600.01	auxin	Hormone signal transduction	Aux/IAA family	7.131864566	2.52881382	0.143579292
FtPinG0001585700.01	auxin	Hormone signal transduction	Aux/IAA family	1.632540183	0.447116519	0.635813728
FtPinG0001961200.01	auxin	Hormone signal transduction	Aux/IAA family	2.294012029	0.174221235	0
FtPinG0003635500.01	auxin	Hormone signal transduction	Aux/IAA family	11.00201	7.872572667	1.151063733
FtPinG0005535200.01	auxin	Hormone signal transduction	Aux/IAA family	3.166618105	0.250047142	0.133203879
FtPinG0001338600.01	auxin	Hormone signal transduction	Aux/IAA family	8.844144446	0.863120879	0
FtPinG0009866800.01	auxin	Hormone signal transduction	Aux/IAA family	0	0	0
FtPinG0007581100.01	auxin	Hormone signal transduction	Aux/IAA family	0.834949983	0.196103447	0.406223725
FtPinG0007414000.01	auxin	Hormone signal transduction	Aux/IAA family	84.59904568	27.73131948	17.42754829
FtPinG0007560600.01	gibberellin	Hormone biosynthesis	iron/ascorbate-dependent oxidoreductase family	2.794776241	0.07857671	0
FtPinG0009540700.01	gibberellin	Hormone biosynthesis	iron/ascorbate-dependent oxidoreductase family	5.894141351	6.871880515	11.51254111
FtPinG0003233400.01	gibberellin	Hormone biosynthesis	iron/ascorbate-dependent oxidoreductase family	4.385205796	12.9933552	0.761198603

FtPinG0008699300.01	gibberellin	Hormone biosynthesis	iron/ascorbate-dependent oxidoreductase family	3.812595443	4.983516698	6.664104505
FtPinG0007802700.01	abscisic acid	Hormone receptor	PYR/PYL/RCAR abscisic acid intracellular receptor family	10.53954818	3.674223001	1.671600433
FtPinG0008671800.01	abscisic acid	Hormone receptor	PYR/PYL/RCAR abscisic acid intracellular receptor family	1.662643433	0.470524587	0.06116484
FtPinG0008047400.01	abscisic acid	Hormone receptor	PYR/PYL/RCAR abscisic acid intracellular receptor family	9.740739819	3.693294221	5.635242686
FtPinG0004424000.01	abscisic acid	Hormone receptor	PYR/PYL/RCAR abscisic acid intracellular receptor family	60.34733726	23.22613274	6.231696795
FtPinG0002423800.01	auxin	Hormone signal transduction	ARG7 family	0.2300927	0.047829479	0.273596611
FtPinG0003158800.01	auxin	Hormone signal transduction	ARG7 family	0.476226127	0.398278551	1.19731728
FtPinG0004309500.01	auxin	Hormone signal transduction	ARG7 family	3.137250816	2.725331486	0.582469781
FtPinG0006187800.01	gibberellin	Hormone signal transduction	GASA family	0.462353841	0.155196752	0.32835851
FtPinG0006187600.01	gibberellin	Hormone signal transduction	GASA family	13.40573939	0.102270648	0.067759873
FtPinG0007619900.01	abscisic acid	Hormone receptor	PYR/PYL/RCAR abscisic acid intracellular receptor family	4.770937515	3.698850475	2.92939277
FtPinG0002102600.01	abscisic acid	Hormone receptor	PYR/PYL/RCAR abscisic acid intracellular receptor family	16.60378756	9.286983264	1.347357692
FtPinG0005799600.01	abscisic acid	Hormone receptor	PYR/PYL/RCAR abscisic acid intracellular receptor family	4.793605888	2.225287967	5.007113984
FtPinG0000874700.01	abscisic acid	Hormone receptor	PYR/PYL/RCAR abscisic acid intracellular receptor family	6.322537782	6.000768487	1.953425945
FtPinG0008891400.01	abscisic acid	Hormone receptor	PYR/PYL/RCAR abscisic acid intracellular receptor family	1.067529322	0.639446166	0.161577557
FtPinG0001407600.01	abscisic acid	Hormone receptor	PYR/PYL/RCAR abscisic acid intracellular receptor family	0.277528103	0.122877965	0.023873502
FtPinG0002387400.01	abscisic acid	Hormone metabolism	cytochrome P450 family	6.708343507	11.79860449	33.42813458
FtPinG0003175900.01	abscisic acid	Hormone metabolism	cytochrome P450 family	10.86337675	11.6477612	26.28104375
FtPinG0005535900.01	auxin	Hormone transportion	auxin efflux carrier (TC 2.A.69.1) family	0.080907444	0	0
FtPinG0005142100.01	auxin	Hormone signal transduction	Aux/IAA family	1.882857141	0.258143407	0.057588734
FtPinG0007967500.01	auxin	Hormone signal transduction	ARG7 family	0.084100171	0	0.221365138
FtPinG0001622200.01	auxin	Hormone signal transduction	ARG7 family	0.180093295	0.19426963	0
FtPinG0003215600.01	auxin	Hormone signal transduction	ARG7 family	3.83924177	5.37311375	2.182016285
FtPinG0001096500.01	auxin	Hormone signal transduction	ARG7 family	0	0	0
FtPinG0007968400.01	auxin	Hormone signal transduction	ARG7 family	0.27719799	0.041378684	0
FtPinG0007966400.01	auxin	Hormone signal transduction	ARG7 family	6.166798469	2.070829217	0
FtPinG0001397900.01	auxin	Hormone signal transduction	ARG7 family	20.06579142	4.977203249	0.48114854
FtPinG0002434500.01	auxin	Hormone signal transduction	ARG7 family	0	0.052917722	0
FtPinG0008444800.01	auxin	Hormone signal transduction	ARG7 family	0.463578764	0.119606584	0
FtPinG0000807600.01	auxin	Hormone signal transduction	ARG7 family	4.644180029	2.805317993	3.86384912
FtPinG0000765000.01	auxin	Hormone signal transduction	ARG7 family	2.052029455	0.769426091	0.076891904
FtPinG0000909000.01	auxin	Hormone signal transduction	ARG7 family	0	0	0

FtPinG0001398200.01	auxin	Hormone signal transduction	ARG7 family	1.886405387	1.160870019	0
FtPinG0009367500.01	auxin	Hormone signal transduction	ARG7 family	0.764600004	0.114379561	0.03627054
FtPinG0005521900.01	auxin	Hormone receptor	NA	5.961210464	3.979247876	2.577627668
FtPinG0007264800.01	auxin	Hormone receptor	NA	7.789059677	3.387215531	1.985086734
FtPinG0006955500.01	auxin	Hormone receptor	NA	30.68487247	37.70174452	64.02700082
FtPinG0006188000.01	gibberellin	Hormone signal transduction	GASA family	21.69803224	24.11764659	4.670413082
FtPinG0002727000.01	gibberellin	Hormone signal transduction	GASA family	263.5074507	150.9166194	117.8664347
FtPinG0007503000.01	gibberellin	Hormone signal transduction	GASA family	12.43454539	11.4833651	0.213963872
FtPinG0006944700.01	gibberellin	Hormone signal transduction	GASA family	3.043448289	3.767206255	0.105735682
FtPinG0000807700.01	auxin	Hormone signal transduction	Aux/IAA family	4.24018152	1.71268214	0.474990247
FtPinG0000764000.01	auxin	Hormone signal transduction	Aux/IAA family	12.67785312	3.277795417	4.381225172
FtPinG0000814000.01	auxin	Hormone signal transduction	Aux/IAA family	0	0	0
FtPinG0008443000.01	auxin	Hormone signal transduction	Aux/IAA family	4.263175276	0.938886554	0.034266715
FtPinG0000464100.01	auxin	Hormone signal transduction	DRM1/ARP family	3986.415024	1015.819875	1893.725417
FtPinG0006959800.01	auxin	Hormone signal transduction	Aux/IAA family	0.770376587	1.191839934	1.403618739
FtPinG0009328100.01	auxin	Hormone signal transduction	Aux/IAA family	0.031502722	0.100100598	0
FtPinG0009166900.01	auxin	Hormone signal transduction	ARG7 family	0	0	0
FtPinG0009169000.01	auxin	Hormone signal transduction	ARG7 family	0.168989685	0	0
FtPinG0009167900.01	auxin	Hormone signal transduction	ARG7 family	0.047585573	0.023685583	0
FtPinG0009164500.01	auxin	Hormone signal transduction	ARG7 family	0.516505432	0.03233566	0
FtPinG0009164300.01	auxin	Hormone signal transduction	ARG7 family	0.421883173	0.358901684	1.570283241
FtPinG0009172200.01	auxin	Hormone signal transduction	ARG7 family	4.684605244	1.629187264	0.676417422
FtPinG0005745300.01	auxin	Hormone signal transduction	Aux/IAA family	0.823686116	0.677205418	0.191503444
FtPinG0008442000.01	auxin	Hormone signal transduction	Aux/IAA family	13.08816361	1.964478377	1.415761657
FtPinG0000809900.01	auxin	Hormone signal transduction	Aux/IAA family	49.39191495	18.98787199	11.5579114
FtPinG0009368700.01	auxin	Hormone signal transduction	Aux/IAA family	120.4628826	47.91497062	15.04652119
FtPinG0007592600.01	auxin	Hormone signal transduction	Aux/IAA family	2.04151817	1.845041048	0.829267055
FtPinG0008025900.01	auxin	Hormone signal transduction	Aux/IAA family	45.13976631	16.27887475	5.524432683
FtPinG0007581000.01	auxin	Hormone signal transduction	Aux/IAA family	0.050424734	0	0
FtPinG0007414500.01	auxin	Hormone signal transduction	Aux/IAA family	5.040388116	1.080208778	0.86949418
FtPinG0002514700.01	gibberellin	Hormone biosynthesis	iron/ascorbate-dependent oxidoreductase family	10.64011993	20.18538513	0.215118003
FtPinG0008198800.01	gibberellin	Hormone biosynthesis	iron/ascorbate-dependent oxidoreductase family	2.230466382	6.940918334	57.17657788
FtPinG0005591800.01	gibberellin	Hormone biosynthesis	iron/ascorbate-dependent oxidoreductase family	2.571535215	0.995191161	0.304392489
FtPinG0004385200.01	gibberellin	Hormone biosynthesis	iron/ascorbate-dependent oxidoreductase family	1.365804251	0.57774693	5.445488563
FtPinG0008005100.01	gibberellin	Hormone biosynthesis	iron/ascorbate-dependent oxidoreductase family	0.035029365	0	0
FtPinG0009397500.01	gibberellin	Hormone biosynthesis	iron/ascorbate-dependent oxidoreductase family	1.553731085	0.833723732	0.286402635
FtPinG0007967400.01	auxin	Hormone signal transduction	ARG7 family	0.42471673	0	0
FtPinG0007968800.01	auxin	Hormone signal transduction	ARG7 family	1.250781043	0.381629448	0

FtPinG0004311700.01	auxin	Hormone transportion	amino acid/polyamine transporter 2 family	0	0	0
FtPinG0001567800.01	auxin	Hormone transportion	auxin efflux carrier (TC 2.A.69.1) family	1.615402495	1.173096637	0.62236154
FtPinG0009541700.01	auxin	Hormone transportion	auxin efflux carrier (TC 2.A.69.1) family	11.32869361	4.04209925	0.635266638
FtPinG0009310400.01	auxin	Hormone transportion	auxin efflux carrier (TC 2.A.69.1) family	0.536173885	0.113202831	0.039931344
FtPinG0005142700.01	auxin	Hormone signal transduction	Aux/IAA family	38.7095567	25.85445665	24.52697318
FtPinG0003898400.01	auxin	Hormone signal transduction	Aux/IAA family	22.0792036	16.74697179	6.617401781
FtPinG0005714900.01	auxin	Hormone signal transduction	Aux/IAA family	1.626601097	2.251144851	2.16619024
FtPinG0002645500.01	auxin	Hormone signal transduction	Aux/IAA family	7.271270825	5.288332216	5.428010126
FtPinG0000437300.01	auxin	Hormone transportion	auxin efflux carrier (TC 2.A.69.1) family	7.771282991	3.007503549	1.127952779
FtPinG0006941400.01	auxin	Hormone transportion	auxin efflux carrier (TC 2.A.69.1) family	2.848346005	1.313839214	0.287798119
FtPinG0002134900.01	cytokinin	Hormone metabolism	oxygen-dependent FAD-linked oxidoreductase family	0.19483645	0.008935807	0
FtPinG0005613800.01	cytokinin	Hormone metabolism	oxygen-dependent FAD-linked oxidoreductase family	7.194924807	7.93101844	6.326682535
FtPinG0002075100.01	gibberellin	Hormone response	GRAS family	65.13253288	15.47588532	15.34979115
FtPinG0007899500.01	gibberellin	Hormone response	GRAS family	1.953996319	1.229839299	1.021018131
FtPinG0007899800.01	gibberellin	Hormone response	GRAS family	2.724043434	1.931748865	1.331439001
FtPinG0001971700.01	auxin	Hormone signal transduction	Aux/IAA family	20.5471059	12.88599884	16.49777708
FtPinG0009169200.01	auxin	Hormone signal transduction	ARG7 family	2.297787962	0.328068276	0.031269374
FtPinG0009165100.01	auxin	Hormone signal transduction	ARG7 family	0.902660588	0.264230608	0
FtPinG0000419200.01	auxin	Hormone signal transduction	ARG7 family	1.786688655	1.023547226	0.067579037
FtPinG0009169300.01	auxin	Hormone signal transduction	ARG7 family	3.362707646	0.2350008	0
FtPinG0009164800.01	auxin	Hormone signal transduction	ARG7 family	12.70407448	10.48226702	15.24643671
FtPinG0009169800.01	auxin	Hormone signal transduction	ARG7 family	0.111254078	0	0
FtPinG0009166700.01	auxin	Hormone signal transduction	ARG7 family	0.390961381	0.068451046	0
FtPinG0009170400.01	auxin	Hormone signal transduction	ARG7 family	0.029741279	0.032092038	0
FtPinG0006350100.01	gibberellin	Hormone signal transduction	GASA family	598.4179449	220.2092626	26.43382362
FtPinG0006417500.01	gibberellin	Hormone signal transduction	GASA family	31.38224256	4.945725403	0.167483954
FtPinG0008597200.01	auxin	Hormone signal transduction	Aux/IAA family	2.188136148	1.182322843	0.887611531
FtPinG0005201900.01	auxin	Hormone signal transduction	Aux/IAA family	26.69772306	10.52058063	6.655092891
FtPinG0005201700.01	auxin	Hormone signal transduction	Aux/IAA family	12.76621549	4.265258104	2.790231161
FtPinG0004139100.01	cytokinin	Hormone biosynthesis	LOG family	9.822419335	5.803127437	3.998483281
FtPinG0007342200.01	cytokinin	Hormone biosynthesis	LOG family	15.2087386	8.343103658	7.672161234
FtPinG0002585600.01	abscisic acid	Hormone receptor	PYR/PYL/RCAR abscisic acid intracellular receptor family	36.77112498	12.52772365	6.570736821
FtPinG0005265100.01	abscisic acid	Hormone receptor	PYR/PYL/RCAR abscisic acid intracellular receptor family	11.32731224	5.754649983	2.890958213
FtPinG0001942600.01	auxin	Hormone signal transduction	Aux/IAA family	0.917284845	0.215017886	0.037225446

FtPinG0000343000.01	auxin	Hormone signal transduction	Aux/IAA family	21.87732928	13.55063048	8.704252769
FtPinG0002610200.01	auxin	Hormone signal transduction	Aux/IAA family	14.54064946	4.104343824	1.560557329
FtPinG0008603100.01	cytokinin	Hormone biosynthesis	LOG family	1.009683697	0.134903521	0.453723421
FtPinG0007288900.01	cytokinin	Hormone biosynthesis	LOG family	14.13962254	15.88338795	6.855083415
FtPinG0001266800.01	cytokinin	Hormone biosynthesis	LOG family	3.281628315	6.460273171	18.05112929
FtPinG0006919600.01	gibberellin	Hormone signal transduction	GASA family	74.035667	8.693631313	0.433217119
FtPinG0001577700.01	auxin	Hormone signal transduction	Aux/IAA family	10.14269196	26.02980159	7.811725015
FtPinG0002311400.01	abscisic acid	Hormone receptor	PYR/PYL/RCAR abscisic acid intracellular receptor family	0.641571415	0.591749935	3.843635492
FtPinG0009506900.01	auxin	Hormone signal transduction	Aux/IAA family	52.63894492	26.41272866	21.29164423
FtPinG0002025600.01	cytokinin	Hormone biosynthesis	LOG family	0.34863507	0.144801292	0.055498084
FtPinG0005927100.01	cytokinin	Hormone biosynthesis	LOG family	0.950519543	0.591516879	0.13247433
FtPinG0003898800.01	cytokinin	Hormone biosynthesis	LOG family	2.77133568	1.143195932	0.169985585
FtPinG0002984800.01	auxin	Hormone signal transduction	Aux/IAA family	26.18979098	13.58115298	13.85030023
FtPinG0004530400.01	auxin	Hormone signal transduction	Aux/IAA family	0.594465959	0.066961404	0.09786756
FtPinG0007273100.01	auxin	Hormone signal transduction	Aux/IAA family	2.304363678	2.858794768	3.664297832
FtPinG0003486500.01	cytokinin	Hormone biosynthesis	LOG family	0.883378876	0.235338637	0.194341807
FtPinG0009006700.01	cytokinin	Hormone biosynthesis	LOG family	0.531501807	0	0.060343594
FtPinG0000536000.01	auxin	Hormone signal transduction	Aux/IAA family	0.393233609	0	0
FtPinG0000683600.01	auxin	Hormone signal transduction	Aux/IAA family	27.61570798	8.42439157	3.253176883
FtPinG0003500800.01	auxin	Hormone signal transduction	Aux/IAA family	14.25974503	8.78172547	10.03986197
FtPinG0003501400.01	auxin	Hormone signal transduction	Aux/IAA family	6.754781824	3.20105742	3.175726536
FtPinG0002445200.01	abscisic acid	Hormone receptor	PYR/PYL/RCAR abscisic acid intracellular receptor family	0.755946391	0.925971647	0.789828282
FtPinG0004588200.01	gibberellin	Hormone receptor	'GDXG' lipolytic enzyme family	40.69748249	15.3625749	15.36089643
FtPinG0009034000.01	abscisic acid	Hormone metabolism	cytochrome P450 family	0.088149922	0.187315225	0.328516882
FtPinG0004478100.01	auxin	Hormone signal transduction	NA	5.826257051	1.852128477	0.725078331
FtPinG0003566300.01	auxin	Hormone signal transduction	NA	19.89803005	20.53384205	3.12265434
FtPinG0002624600.01	auxin	Hormone signal transduction	NA	1.121969892	1.191412692	0.450210774
FtPinG0002469700.01	auxin	Hormone signal transduction	Aux/IAA family	29.09579493	14.01585713	11.27409353
FtPinG0005575800.01	auxin	Hormone signal transduction	Aux/IAA family	81.0331684	28.09909973	17.12383403
FtPinG0009165500.01	auxin	Hormone signal transduction	ARG7 family	0	0	0
FtPinG0009165400.01	auxin	Hormone signal transduction	ARG7 family	0	0	0
FtPinG0009163900.01	auxin	Hormone signal transduction	ARG7 family	0.971403437	0.398526764	0.208931897
FtPinG0009163500.01	auxin	Hormone signal transduction	ARG7 family	0.328669629	0.167340696	0.032435034
FtPinG0009167700.01	auxin	Hormone signal transduction	ARG7 family	1.319128374	0.132583244	0
FtPinG0009164900.01	auxin	Hormone signal transduction	ARG7 family	0	0	0
FtPinG0009171800.01	auxin	Hormone signal transduction	ARG7 family	0.115801421	0	0
FtPinG0009167300.01	auxin	Hormone signal transduction	ARG7 family	1.303011333	0.111817208	0
FtPinG0009171400.01	auxin	Hormone signal transduction	ARG7 family	0	0	0
FtPinG0006908400.01	auxin	Hormone transportion	amino acid/polyamine transporter 2 family	49.75627658	4.480345984	0.225637095
FtPinG0003888700.01	auxin	Hormone transportion	amino acid/polyamine transporter 2 family	19.17579691	3.732455437	2.523907063

FtPinG0004311300.01	auxin	Hormone transportion	amino acid/polyamine transporter 2 family	3.615070287	0.809430426	0.576991366
FtPinG0006532300.01	auxin	Hormone transportion	amino acid/polyamine transporter 2 family	4.044084277	3.358311186	4.760408882
FtPinG0005765500.01	auxin	Hormone transportion	amino acid/polyamine transporter 2 family	90.2986145	69.29121839	89.26665155
FtPinG0007484100.01	cytokinin	Hormone biosynthesis	cytochrome P450 family	0	0	0
FtPinG0007828000.01	cytokinin	Hormone biosynthesis	cytochrome P450 family	0.298692243	0	0
FtPinG0003093300.01	auxin	Hormone transportion	auxin efflux carrier (TC 2.A.69.1) family	0.158182189	0.195534985	0.07524928
FtPinG0005001000.01	auxin	Hormone transportion	auxin efflux carrier (TC 2.A.69.1) family	0.022671631	0	0
FtPinG0004913500.01	auxin	Hormone transportion	auxin efflux carrier (TC 2.A.69.1) family	5.022181466	7.015916014	1.06435871
FtPinG0007479900.01	auxin	Hormone transportion	auxin efflux carrier (TC 2.A.69.1) family	0	0	0
FtPinG0001214600.01	abscisic acid	Hormone receptor	PYR/PYL/RCAR abscisic acid intracellular receptor family	79.59006688	40.30912738	11.135144
FtPinG0005737100.01	auxin	Hormone signal transduction	Aux/IAA family	21.08179056	6.660483404	2.988411791
FtPinG0005737300.01	auxin	Hormone signal transduction	Aux/IAA family	40.64362244	12.78135583	5.920377769
FtPinG0004636500.01	auxin	Hormone signal transduction	Aux/IAA family	15.59065292	5.576533647	8.596930777
FtPinG0004462100.01	abscisic acid	Hormone metabolism	cytochrome P450 family	52.77529124	3.388359326	4.302804152
FtPinG0001623000.01	auxin	Hormone signal transduction	ARG7 family	0.602575563	1.010200587	1.997765584
FtPinG0001623200.01	auxin	Hormone signal transduction	ARG7 family	0.246229889	0	0
FtPinG0007969000.01	auxin	Hormone signal transduction	ARG7 family	2.174381214	0.249264141	0
FtPinG0007968100.01	auxin	Hormone signal transduction	ARG7 family	7.135062734	1.999268103	0.042255911
FtPinG0001621800.01	auxin	Hormone signal transduction	ARG7 family	0.205430107	0.252953425	0.211288224
FtPinG0001621900.01	auxin	Hormone signal transduction	ARG7 family	2.992186884	0.568680385	0
FtPinG0002508600.01	auxin	Hormone signal transduction	Aux/IAA family	0.892915301	0.481120576	0.323675413
FtPinG0003101700.01	cytokinin	Hormone metabolism	oxygen-dependent FAD-linked oxidoreductase family	14.67303667	6.030512419	3.040411466
FtPinG0004375600.01	auxin	Hormone transportion	NA	21.53232422	9.019908015	3.612217758
FtPinG0003072700.01	abscisic acid	Hormone signal transduction	bZIP family	0.401806567	0.15551333	0.237237915
FtPinG0002063700.01	abscisic acid	Hormone signal transduction	bZIP family	5.420195721	3.3578717	0.263233989
FtPinG0009718300.01	abscisic acid	Hormone signal transduction	bZIP family	3.902089939	2.155713688	1.274747909
FtPinG0008174200.01	abscisic acid	Hormone signal transduction	bZIP family	15.93703638	14.35744758	2.374686328
FtPinG0006458700.01	abscisic acid	Hormone signal transduction	bZIP family	14.86759975	18.09813167	26.57380624
FtPinG0007855800.01	gibberellin	Hormone signal transduction	GASA family	32.22946024	45.42557803	91.96956411
FtPinG0002378800.01	abscisic acid	Hormone metabolism	cytochrome P450 family	1.419267665	0.938798717	0.247596499
FtPinG0003013300.01	abscisic acid	Hormone metabolism	cytochrome P450 family	5.440399773	2.449426093	1.699499025
FtPinG0003196900.01	abscisic acid	Hormone metabolism	cytochrome P450 family	6.314648715	3.266957442	2.301156508
FtPinG0003614600.01	abscisic acid	Hormone metabolism	cytochrome P450 family	6.413476375	2.712269057	0.134004571
FtPinG0008345600.01	cytokinin	Hormone metabolism	oxygen-dependent FAD-linked oxidoreductase family	10.82997621	5.348346792	1.913373534

FtPinG0005727300.01	auxin	Hormone signal transduction	ARG7 family	0.196583704	0.347992783	0.194892823
FtPinG0005124800.01	auxin	Hormone signal transduction	ARG7 family	0.128389143	0	0
FtPinG0005727600.01	auxin	Hormone signal transduction	ARG7 family	0	0	0
FtPinG0002423200.01	auxin	Hormone signal transduction	ARG7 family	0.140093755	0	0.18128775
FtPinG0007132900.01	auxin	Hormone transportion	auxin efflux carrier (TC 2.A.69.1) family	0.494532225	1.081663429	1.141349882
FtPinG0001537500.01	cytokinin	Hormone metabolism	oxygen-dependent FAD-linked oxidoreductase family	1.941162598	1.433864029	2.090959079
FtPinG0005658800.01	cytokinin	Hormone metabolism	oxygen-dependent FAD-linked oxidoreductase family	0.995251438	0.937495133	0.065545458
FtPinG0005053900.01	gibberellin	Hormone receptor	'GDXG' lipolytic enzyme family	21.14651605	15.0733684	13.2303805
FtPinG0007990400.01	gibberellin	Hormone receptor	'GDXG' lipolytic enzyme family	20.93306114	8.32169543	5.580790573
FtPinG0006952100.01	gibberellin	Hormone receptor	'GDXG' lipolytic enzyme family	10.68008894	1.314417569	0.631179864
FtPinG0002143600.01	abscisic acid	Hormone signal transduction	bZIP family	29.19396229	4.257546684	0.639857061
FtPinG0009765500.01	abscisic acid	Hormone signal transduction	bZIP family	17.02964704	8.363920222	2.776050991
FtPinG0001379200.01	abscisic acid	Hormone signal transduction	bZIP family	24.10759337	17.53864105	0.100644494
FtPinG0003012600.01	abscisic acid	Hormone signal transduction	bZIP family	15.50835408	11.25496959	26.72565309
FtPinG0003196200.01	abscisic acid	Hormone signal transduction	bZIP family	10.49802894	17.89273521	67.94908992
FtPinG0007662200.01	auxin	Hormone transportion	auxin efflux carrier (TC 2.A.69.1) family	0.437887683	0.094906411	0.036410003
FtPinG0009346000.01	auxin	Hormone transportion	auxin efflux carrier (TC 2.A.69.1) family	9.136154526	3.914110181	3.525818324
FtPinG0009310600.01	auxin	Hormone transportion	auxin efflux carrier (TC 2.A.69.1) family	0	0	0
FtPinG0007643300.01	auxin	Hormone transportion	amino acid/polyamine transporter 2 family	10.06562593	1.758528382	0.299743126
FtPinG0007044300.01	auxin	Hormone signal transduction	ARG7 family	0.096875266	0.17326591	0.438539495
FtPinG0002808700.01	auxin	Hormone signal transduction	ARG7 family	22.84968022	23.11425614	26.36644646
FtPinG0000908700.01	auxin	Hormone signal transduction	ARG7 family	0.027297151	0	0
FtPinG0002403100.01	auxin	Hormone signal transduction	ARG7 family	0	0.025990126	0
FtPinG0007172700.01	auxin	Hormone signal transduction	ARG7 family	0.995323726	5.322621677	2.679213871
FtPinG0003159200.01	auxin	Hormone signal transduction	ARG7 family	0.172921531	0.878290912	0.036643798
FtPinG0008563700.01	auxin	Hormone signal transduction	ARG7 family	0	0	0
FtPinG0001657300.01	auxin	Hormone signal transduction	ARG7 family	0	0.036703613	0
FtPinG0008667300.01	auxin	Hormone signal transduction	ARG7 family	0	0	0
FtPinG0007504400.01	auxin	Hormone signal transduction	ARG7 family	0.429759075	0.25920045	0.725413876
FtPinG0003159000.01	auxin	Hormone signal transduction	ARG7 family	9.490126073	7.743071719	5.859976308
FtPinG0002465500.01	auxin	Hormone signal transduction	ARG7 family	0	0	0
FtPinG0003523300.01	abscisic acid	Hormone signal transduction	bZIP family	28.43375008	65.40430468	162.9254378
FtPinG0000249600.01	gibberellin	Hormone biosynthesis	iron/ascorbate-dependent oxidoreductase family	39.16192323	20.68848287	27.93482637
FtPinG0007406000.01	gibberellin	Hormone biosynthesis	iron/ascorbate-dependent oxidoreductase family	12.03949851	6.001086222	58.5042227
FtPinG0005537400.01	auxin	Hormone signal transduction	UBR4 family	14.71236778	8.445230159	7.251596288

FtPinG0005537600.01	auxin	Hormone signal transduction	UBR4 family	3.052451129	1.978731886	0.154901229
FtPinG0001799400.01	abscisic acid	Hormone receptor	PYR/PYL/RCAR abscisic acid intracellular receptor family	14.74442759	15.85266421	5.297746207
FtPinG0008597700.01	auxin	Hormone signal transduction	Aux/IAA family	36.7422984	41.92382883	75.65326958
FtPinG0002330600.01	gibberellin	Hormone biosynthesis	iron/ascorbate-dependent oxidoreductase family	0.606954286	2.234854474	0.937254048
FtPinG0006464400.01	gibberellin	Hormone biosynthesis	iron/ascorbate-dependent oxidoreductase family	0	0	0.052653484
FtPinG0005929600.01	gibberellin	Hormone biosynthesis	iron/ascorbate-dependent oxidoreductase family	0.072809066	0.113145048	0.281571415
FtPinG0000400800.01	gibberellin	Hormone biosynthesis	iron/ascorbate-dependent oxidoreductase family	0.085644557	0.039435262	0.038340615
FtPinG0009147800.01	gibberellin	Hormone biosynthesis	iron/ascorbate-dependent oxidoreductase family	0	0	0
FtPinG0000387700.01	auxin	Hormone signal transduction	Aux/IAA family	15.7810233	12.65622136	28.43500408
FtPinG0001741700.01	auxin	Hormone signal transduction	Aux/IAA family	34.26929913	19.37470096	22.59248466
FtPinG0002591700.01	auxin	Hormone signal transduction	Aux/IAA family	29.11174521	8.948655388	10.25872118
FtPinG0008266700.01	auxin	Hormone receptor	germin family	59.59093143	8.800831325	0.473339837
FtPinG0005922500.01	auxin	Hormone receptor	germin family	1.914848815	0.271810065	0
FtPinG0005029300.01	auxin	Hormone signal transduction	Aux/IAA family	15.45897662	6.080765125	1.338302781
FtPinG0002499600.01	auxin	Hormone signal transduction	Aux/IAA family	14.94576494	3.838702815	0.62278962
FtPinG0000840000.01	auxin	Hormone signal transduction	Aux/IAA family	27.71730892	12.56428161	11.819527
FtPinG0003331700.01	auxin	Hormone signal transduction	Aux/IAA family	27.90685361	19.97732232	2.263872647
FtPinG0004040700.01	auxin	Hormone signal transduction	Aux/IAA family	8.257506759	2.949521296	1.117617853
FtPinG0006935800.01	auxin	Hormone signal transduction	ARG7 family	1.996254329	0.338814471	0.376811821
FtPinG0002594300.01	auxin	Hormone signal transduction	ARG7 family	123.8087971	119.1572716	23.07200127
FtPinG0004378100.01	auxin	Hormone signal transduction	ARG7 family	29.20351075	51.26464217	8.96800298
FtPinG0001589600.01	auxin	Hormone signal transduction	ARG7 family	59.26996807	24.37239161	19.44738562
FtPinG0002363800.01	auxin	Hormone signal transduction	ARG7 family	71.85674096	139.5890649	88.76974528
FtPinG0008308300.01	cytokinin	Hormone biosynthesis	cytochrome P450 family	1.331980909	2.136398633	0.082684299
Novel00946	auxin	Hormone receptor	NA	10.82226382	4.579957561	2.627875482
Novel01990	auxin	Hormone signal transduction	Aux/IAA family	7.313016883	4.646133445	5.124606678
Novel01557	auxin	Hormone signal transduction	Aux/IAA family	0.020867657	0.040920418	0
Novel02020	auxin	Hormone signal transduction	Aux/IAA family	11.44922251	14.82044618	15.83512033
Novel01693	auxin	Hormone signal transduction	Aux/IAA family	3.174671093	1.729209443	1.410115873
Novel00986	auxin	Hormone signal transduction	UBR4 family	1.701528551	1.1327087	1.085303326

Table S5 List of genes were related to flavonoid during seed development.

Gene ID	Related	Function category	Family	X2 fpkm	X3 fpkm	X4 fpkm
FtPinG0003900700.01	PAL	Phenylpropanoid metabolism	PAL/histidase family	76.28942	29.19908	10.02819
FtPinG0008236900.01	PAL	Phenylpropanoid metabolism	PAL/histidase family	61.09623	93.32882	49.74058
FtPinG0008237100.01	PAL	Phenylpropanoid metabolism	PAL/histidase family	7.947951	3.047234	1.390171
FtPinG0008741700.01	CHS	Flavonoid biosynthesis	chalcone/stilbene synthases family	0	0	0
FtPinG0002106500.01	CHS	Flavonoid biosynthesis	chalcone/stilbene synthases family	0.346682	0.440283	0.603202
FtPinG0008131000.01	CHS	Flavonoid biosynthesis	chalcone/stilbene synthases family	200.6596	265.0281	24.32931
FtPinG0008806400.01	CHS	Flavonoid biosynthesis	chalcone/stilbene synthases family	107.7812	88.55083	19.0516
FtPinG0002790600.01	CHI	Flavonoid biosynthesis	chalcone isomerase family	65.46905	50.18828	7.609164
FtPinG0008145300.01	CHI	Flavonoid biosynthesis	chalcone isomerase family	0.184093	0.058521	0.038933
FtPinG0001742500.01	FLS	Flavonoid biosynthesis	iron/ascorbate-dependent oxidoreductase family	0	0	0
FtPinG0006907100.01	FLS	Flavonoid biosynthesis	iron/ascorbate-dependent oxidoreductase family	335.0774	389.0795	77.87107
FtPinG0004192700.01	FLS	Flavonoid biosynthesis	iron/ascorbate-dependent oxidoreductase family	0.73719	0.691101	0.146988
FtPinG0001742000.01	FLS	Flavonoid biosynthesis	iron/ascorbate-dependent oxidoreductase family	0.287567	0.104861	1.572384
FtPinG0001318800.01	FLS	Flavonoid biosynthesis	iron/ascorbate-dependent	7.889077	5.01474	0.471684

			oxidoreductase family			
FtPinG0001317600.01	FLS	Flavonoid biosynthesis	iron/ascorbate-dependent oxidoreductase family	0	0	0
FtPinG0006907000.01	FLS	Flavonoid biosynthesis	iron/ascorbate-dependent oxidoreductase family	38.57619	18.90718	2.625413
FtPinG0008448800.01	FLS	Flavonoid biosynthesis	iron/ascorbate-dependent oxidoreductase family	0.950497	4.239906	2.541538
FtPinG0003632300.01	Flavonoid 3',5'-hydroxylase	anthocyanin biosynthesis	cytochrome P450 family	0	0.048827	0.193342
FtPinG0003628700.01	Flavonoid 3',5'-hydroxylase	anthocyanin biosynthesis	cytochrome P450 family	0.402304	0	0
FtPinG0003631500.01	Flavonoid 3',5'-hydroxylase	anthocyanin biosynthesis	cytochrome P450 family	0	0	0
FtPinG0006940000.01	Flavonoid 3',5'-hydroxylase	anthocyanin biosynthesis	cytochrome P450 family	14.74328	14.16386	4.213059
FtPinG0003628500.01	Flavonoid 3',5'-hydroxylase	anthocyanin biosynthesis	cytochrome P450 family	3.364915	1.557404	0.094356
FtPinG0007326600.01	UDP-glucose flavonoid 3-O-glucosyltransferase	metabolic process	UDP-glycosyltransferase family	0.745266	2.188527	3.302759
FtPinG0001031900.01	UDP-glucose flavonoid 3-O-glucosyltransferase	metabolic process	UDP-glycosyltransferase family	2.927817	2.598172	8.87172
FtPinG0003187300.01	UDP-glucose flavonoid 3-O-glucosyltransferase	metabolic process	UDP-glycosyltransferase family	3.658782	3.775185	22.16688
FtPinG0001031700.01	UDP-glucose flavonoid 3-O-glucosyltransferase	metabolic process	UDP-glycosyltransferase family	34.4875	42.37771	74.83619
FtPinG0003584500.01	UDP-glucose flavonoid 3-O-glucosyltransferase	metabolic process	UDP-glycosyltransferase family	5.376592	4.342541	0.703926
FtPinG0002704400.01	UDP-glucose flavonoid 3-O-glucosyltransferase	metabolic process	UDP-glycosyltransferase family	4.074893	3.256418	1.369774
FtPinG0007577000.01	UDP-glucose flavonoid 3-O-glucosyltransferase	metabolic process	UDP-glycosyltransferase family	0.254487	0.133343	0.220311
FtPinG0002956600.01	UDP-glucose flavonoid 3-O-glucosyltransferase	metabolic process	UDP-glycosyltransferase family	1.538151	0.113106	1.74673
FtPinG0003585500.01	UDP-glucose flavonoid	metabolic process	UDP-glycosyltransferase	1.707956	3.568884	18.53741

	3-O-glucosyltransferase		family			
FtPinG0003186500.01	UDP-glucose flavonoid 3-O-glucosyltransferase	metabolic process	UDP-glycosyltransferase family	7.891746	4.365215	0.889886
FtPinG0006615400.01	UDP-glucose flavonoid 3-O-glucosyltransferase	metabolic process	UDP-glycosyltransferase family	10.31112	17.67281	11.56381
FtPinG0003186900.01	UDP-glucose flavonoid 3-O-glucosyltransferase	metabolic process	UDP-glycosyltransferase family	0.010209	0	0
FtPinG0003585700.01	UDP-glucose flavonoid 3-O-glucosyltransferase	metabolic process	UDP-glycosyltransferase family	3.370236	2.244972	0.785647
FtPinG0003585900.01	UDP-glucose flavonoid 3-O-glucosyltransferase	metabolic process	UDP-glycosyltransferase family	1.17426	0.071696	0.008675
FtPinG0003629500.01	Flavonoid 3'-monooxygenase	anthocyanin biosynthesis	cytochrome P450 family	1.55294	2.792627	6.289344
FtPinG0002353900.01	Flavonoid 3'-monooxygenase	flavonoid biosynthesis	cytochrome P450 family	54.95068	33.46139	14.36979
FtPinG0001470800.01	Flavonoid 3'-monooxygenase	flavonoid biosynthesis	cytochrome P450 family	0	0.009248	0
FtPinG0008925900.01	Flavonoid 3'-monooxygenase	flavonoid biosynthesis	cytochrome P450 family	0	0	0
FtPinG0003630100.01	Flavonoid 3'-monooxygenase	flavonoid biosynthesis	cytochrome P450 family	0.020337	0.010122	0.123129
FtPinG0003629700.01	Flavonoid 3'-monooxygenase	flavonoid biosynthesis	cytochrome P450 family	5.429075	6.165407	11.73077
FtPinG0001470600.01	Flavonoid 3'-monooxygenase	flavonoid biosynthesis	cytochrome P450 family	6.173626	11.31342	3.404723
FtPinG0003632500.01	Flavonoid 3'-monooxygenase	flavonoid biosynthesis	cytochrome P450 family	0.12381	0	0.02524
FtPinG0001471000.01	Flavonoid 3'-monooxygenase	flavonoid biosynthesis	cytochrome P450 family	0.025532	0.045975	0
FtPinG0003628300.01	Flavonoid 3'-monooxygenase	flavonoid biosynthesis	cytochrome P450 family	0	0	0
FtPinG0003630300.01	Flavonoid 3'-monooxygenase	flavonoid biosynthesis	cytochrome P450 family	0.056159	0	0.027987

Table S6 Validation of the transcriptome data by qRT-PCR.

GeneID	X2_fpkm	X3_fpkm	X4_fpkm	X2_relative expression	X3_relative expression	X4_relative expression
FtPinG0000494800.01(*FtAGL9*)	31.816	15.209	6.792	11.567	3.600	1.019
FtPinG0002495300.01(*FtAGL*)	332.842	127.130	16.869	47.840	5.776	1.007
FtPinG0003934400.01(*FtGAIL*)	15.131	10.369	1.741	42.623	13.902	1.050
FtPinG0004500000.01(*FtSEP1*)	26.014	15.114	5.850	7.592	1.819	1.006
FtPinG0004510400.01(*FtSEP1L*)	120.021	64.465	11.890	15.015	3.271	1.087
FtPinG0005096000.01(*FtSEP1-2*)	47.541	9.668	1.143	8.480	2.246	1.058
FtPinG0005485500.01(*FtSEP3L*)	2.772	2.265	1.776	1.013	0.196	0.003
FtPinG0006608000.01(*FtSEP1L-2*)	100.210	78.249	21.659	141.373	16.118	1.004
FtPinG0003900700.01(*FtPAL*)	76.289	29.199	10.028	1.009	0.814	0.389
FtPinG0008131000.01(*FtCHS*)	200.660	265.028	24.329	11.809	19.677	1.011
FtPinG0002790600.01(*FtCHI*)	65.469	50.188	7.609	16.895	8.093	1.003
FtPinG0006907100.01(*FtFLS*)	335.077	389.079	77.871	2.475	3.982	1.014
FtPinG0006606900.01(*FtUFGT*)	128.934	91.673	67.730	2.019	1.976	1.000

Table S7 Primers of sequences.

**Gene ID**	**Forward primer (5`- 3`)**	**Reverse primer (5`- 3`)**
FtPinG0000494800.01(*FtAGL9*)	ACGAACCCAGGTCACGAAATC	AAGCCACGCACCAGGAATG
FtPinG0002495300.01(*FtAGL*)	GCCCAGTATTATCAGCAAGAAGC	GCTACTGAGCCCTTCACCCAT
FtPinG0003934400.01(*FtGAIL*)	ACCGCCAATCAAGCGATACTC	TGAGTGCAAGGGCTTGGATTA
FtPinG0004500000.01(*FtSEP1*)	TGCAAAGAGGAGAAATGGCC	GCTGGTGCTGGAGAACTCATAGA
FtPinG0004510400.01(*FtSEP1L*)	GGTCATCAACAACATCCTCAATCTC	CACTTGACCCACATTTGTAGTTTGA
FtPinG0005096000.01(*FtSEP1-2*)	GCAATGTAACAGCAGCCTTCAA	AGCAACCACTCAGTCGCAAAC
FtPinG0005485500.01(*FtSEP3L*)	ATTCCTCACGCAAATTATCACCTC	CAGCTCTTTCATGCTTAAACTCGA
FtPinG0006608000.01(*FtSEP1L-2*)	AAGATGCTGAAGATGCTGACAACTC	CATAGCCATGTGACTGTAACCGA
FtPinG0003900700.01(*FtPAL*)	AGATCAACTCCGTCAACGATAACC	AGCTCTGAGAATTGGGCAAACA
FtPinG0008131000.01(*FtCHS*)	GATTGCTCATCCTGGTGGTCC	CGATTTCTTCCTCATCTCGTCCAT
FtPinG0002790600.01(*FtCHI*)	GCTGCTCGACTCTATGAACTGCT	ATGGCTATGGAGGATACGGTAATT
FtPinG0006907100.01(*FtFLS*)	CCAATGCTCTCATCGTTCACGT	CAAGAACACAGGCCACGACATC
FtPinG0006606900.01(*FtUFGT*)	TGGCTGATGCCTTTCTGTCG	AGTCTTCATAGTGTGCTTCTCCGG
FtPinG0002353900.01(*FtF3'H*)	GAGATTTGACAGCTTTATCGGACAG	AAGAGCAGAGCCTTGATTTCAGTAT
FtPinG0003628500.01(*FtF3'5'H*)	ATGGAGGATGCTACGAAAGGTCT	CGTCACCACGTTCATTGCTGTT
FtPinG0005072700.01(*Ft4CL*)	TAATGGTGAGGTTCCTGTGGCA	CGGAATTGCGTGAACGAAGTAC
FtPinG0001575100.01(*FtC4H*)	AATCTCGTGGTCGTCTCGTCA	GTCATTATCCGCCGCATCTT
FtPinG0000381200.01(*FtNST1*)	CGATTTAGGAGGAGATGAAGGGT	GAGTCGAGTTTCGGTTGTTGGT
FtPinG0002596000.01(*FtNST1-2*)	CATGCTTGCCGCTCACTTAAAC	TGCGGTGATGGACGGGTAGTA
FtPinG0007471500.01(*FtNST2*)	AGCTTCAACCGCGAACTGG	GACTCACCGTATGCTCCTCTGG
FtH3	GAAATTCGCAAGTACCAGAAGAG	CCAACAAGGTATGCCTCAGC
